# Periostin gene expression in neu-positive breast cancer cells is regulated by a FGFR signaling cross talk with TGFβ/PI3K/AKT pathways

**DOI:** 10.1186/s13058-021-01487-8

**Published:** 2021-11-22

**Authors:** Cédrik Labrèche, David P. Cook, John Abou-Hamad, Julia Pascoal, Benjamin R. Pryce, Khalid N. Al-Zahrani, Luc A. Sabourin

**Affiliations:** 1 Centre for Cancer Therapeutics, Ottawa Hospital Research Institute, 501 Smyth Road, Ottawa, K1H 8L6 ON UK; 2 Department of Cellular and Molecular Medicine, University of Ottawa, Ottawa, ON K1H 8M5 Canada; 3 Present Address: Department of Pediatrics, Hollings Cancer Center, Medical University of South Carolina, Charleston, SC 29425 USA; 4 Present Address: Lunenfeld-Tanenbaum Research Institute, 600 University Avenue, Toronto, ON M5G 1X5 Canada

**Keywords:** Periostin, Gene regulation, Breast cancer, FGF, AKT

## Abstract

**Background:**

Breast cancer is a highly heterogeneous disease with multiple drivers and complex regulatory networks. Periostin (Postn) is a matricellular protein involved in a plethora of cancer types and other diseases. Postn has been shown to be involved in various processes of tumor development, such as angiogenesis, invasion, cell survival and metastasis. The expression of Postn in breast cancer cells has been correlated with a more aggressive phenotype. Despite extensive research, it remains unclear how epithelial cancer cells regulate Postn expression.

**Methods:**

Using murine tumor models and human TMAs, we have assessed the proportion of tumor samples that have acquired Postn expression in tumor cells. Using biochemical approaches and tumor cell lines derived from Neu+ murine primary tumors, we have identified major regulators of Postn gene expression in breast cancer cell lines.

**Results:**

Here, we show that, while the stromal compartment typically always expresses Postn, about 50% of breast tumors acquire Postn expression in the epithelial tumor cells. Furthermore, using an in vitro model, we show a cross-regulation between FGFR, TGFβ and PI3K/AKT pathways to regulate Postn expression. In HER2-positive murine breast cancer cells, we found that basic FGF can repress Postn expression through a PKC-dependent pathway, while TGFβ can induce Postn expression in a SMAD-independent manner. Postn induction following the removal of the FGF-suppressive signal is dependent on PI3K/AKT signaling.

**Conclusion:**

Overall, these results reveal a novel regulatory mechanism and shed light on how breast tumor cells acquire Postn expression. This complex regulation is likely to be cell type and cancer specific as well as have important therapeutic implications.

**Supplementary Information:**

The online version contains supplementary material available at 10.1186/s13058-021-01487-8.

## Background

Excluding non-melanoma skin cancer, breast cancer is the most common type of cancer in women worldwide [1]. Breast cancer is a complex and highly heterogeneous disease which is typically classified in molecular subtypes according to the expression of specific factors such as estrogen receptor (ESR1), progesterone receptor (PGR) and human epidermal growth factor receptor 2 (HER2) [2, 3]. The HER2-positive subtype is characterized by an overexpression of HER2 with low or no expression of ESR1 and PGR. The prevalence of this subtype is approximately 25–30% and is strongly associated with highly metastatic disease and a poor prognosis [4]. Although the subtypes are generally well characterized, the molecular mechanisms of HER2-mediated tumorigenesis are not completely understood. Multiple genetic and biochemical alterations have been shown to contribute to the progression and metastasis of breast cancer regardless of their subtype. In addition to oncogenes, inflammatory pathways have also been demonstrated to recruit immune cells and contribute to changes in the tumor microenvironment.

Periostin (Postn), also known as osteoblast-specific factor 2 (OSF-2) [5], is a 90 kDa secreted matricellular protein containing an EMI domain (named after the EMILIN family of protein), involved in protein–protein interactions, four tandem repeats of fasciclin-like domains (FAS1 domains) known to interact with integrins and a C-terminal region mediating interactions with other members of the extracellular matrix such as tenascin-C [6] and bone morphogenetic protein 1 (BMP-1) [7, 8]. These interactions make Postn a key regulator of cell behavior and extracellular matrix remodeling. More specifically, its interactions with integrin αvβ3 and αvβ5 have been linked to the activation of AKT, PI3K and FAK signaling pathways in multiple cell types including osteoblasts, normal and cancer cells [9–11]. The main role of Postn in healthy tissue appears to be binding to BMP-1 and collagen, therefore acting as a scaffold to accelerate collagen cross-linking by promoting proteolytic activation of lysyl oxidase (LOX) [7]. Supporting this, knockout animals display aberrant collagen fibrillogenesis [12] and a reduction in collagen cross-linking in skin, tendons and cardiac tissue [13].

Postn gene regulation has been extensively studied in a plethora of models. Multiple genes have been identified as potential regulators, but evidence shows that it is strongly context dependent. For example, it has been shown that some classical epithelial-to-mesenchymal transition (EMT) triggers such as TGFβ and Twist are able to promote Postn expression in osteoblastic lineages [8, 14]. Furthermore, several cytokines, growth factors and hormones have also been identified as inducers of Postn expression in different animal models and cell types. For instance, bFGF, IL-4, IL-13, oncostatin M, PDGF and TNFα have all been shown to induce Postn expression in various systems [15–19]. Although its expression is significantly correlated with poor prognosis and metastasis [20–23], the regulation of Postn gene expression in breast cancer is poorly understood.

Postn overexpression has been correlated with several cancer-driving mechanisms such as cell survival, migration, angiogenesis, EMT and metastasis through its binding to members of the extracellular matrix and integrin receptors [9, 24]. Although Postn is mostly expressed in fibroblasts in normal mammary tissues, acquired expression by cancer cells has been correlated with poor prognosis and survival in breast cancer patients, making Postn expression by epithelial cells of prognostic value [20, 25]. A study showed that 57.7% of invasive breast carcinomas have a high epithelial Postn expression, while 83.6% have high Postn stromal expression [20].

A large body of evidence suggests that basic fibroblast growth factor (bFGF; FGF2)/FGF receptor signaling is critical for multiple biological functions such as embryonic development, wound repair, tissue regeneration and normal hematopoiesis [26]. Basic FGF is the prototype member of the FGF family containing 23 FGF signaling ligands and the most extensively studied member as well [27]. It can form a complex along with heparan sulfate proteoglycans (HSPGs) and FGFRs to activate Ras, Raf, MAPK and ERK [28]. Basic FGF has also been identified as an activator of PI3K/AKT in multiple models and tissues [29, 30] and has been classified as mitogenic for tumor cells and stromal cells altogether due to its wide effects on intracellular signaling [31, 32]. Regulation of bFGF and FGFR is highly complex, and receptor internalization is the main signaling termination mechanism [26].

As previously reported [25, 33], here we show that 40–50% of human breast cancers acquire Postn expression in epithelial cancer cells. Similarly, acquired Postn expression is observed at the same frequency in three different murine models of HER2+ breast cancers [34–36]. To gain insights into the mechanisms regulating Postn expression in breast cancer cells, we established an in vitro model of acquired Postn expression to identify potential signaling pathways regulating its expression. In vitro analyses showed that Postn expression is repressed by bovine pituitary extract, a component of mammary epithelial cell culture medium. Further analyses showed that bFGF is the repressive activity in the culture medium. Removal of bFGF results in Postn induction that is dependent on PI3K/Akt and TGFβ, but independent of Smad2. Together, our data suggest that bFGF can activate a repressive cross talk to PI3K/Akt, downstream of TGFβ receptor signaling to regulate Postn expression in breast cancer cells.

## Methods

### Animals

MMTV-NeuNDL [34], MMTV-NIC [35] and MMTV-PyMT [36] transgenic mice were a kind gift from Dr. William Muller (McGill University). Postn-null mice [37] were a kind gift from Dr. Simon J. Conway. Female mice only were used for all experiments. Mice were palpated twice a week to assess onset, progression and endpoint. Tumor onset was defined by a volume of 0.5 cm^3^, whereas endpoint tumors were defined by a total tumor burden of 1.7 cm^3^. To determine the size of the tumor, the longest diameter of the tumor was used to calculate a spherical volume (*V* = ¾*πr*^3^). All mouse experiments were conducted following Animal Care and Veterinary Services guidelines and standards at the University of Ottawa.

### Analysis of triple-negative scRNA-seq data

Single-cell RNA-seq of five TNBC tumors was originally performed by Wu et al. [38]*.* Raw UMI counts and cell metadata were acquired from ENA accession PRJEB35405. The data were processed with Seurat v4.0.0 [39]. Normalization and feature selection were performed using SCTransform [40] while regressing out the proportion of mitochondrial reads. Normalized data were then processed with principal component analysis prior to generating UMAP embeddings based on the first 30 PCs. We then partitioned the cells using Louvain-based clustering on the first 30 PCs using the FindNeighbors(dims = 1:30) and FindClusters(resolution = 0.2) functions implemented in Seurat. Cell-type annotations from the original publication were used to define cluster labels for the data. All expression data shown in figures correspond to log-transformed counts per 10,000 UMIs.

Signaling pathway activity inference was performed with PROGENy (v1.11.2) [41], which infers activity based on pre-built models of pathway effects based from collections of perturbation experiments. Scores were calculated based on the normalized expression values for each cell using the progeny function with top = 500 to base scores on the top 500 genes of its models. Activity scores correspond to Z-score transformations, and the figures presented only show values > 0 to only highlight cells with higher-than-average levels of pathway activity.

### Cell culture and primary cell isolation

Tumors from MMTV-NeuNDL mice or Postn −/−:NeuNDL were harvested at endpoint, minced and digested as previously described [42]. Murine breast cancer lines were maintained in DMEM/F12 containing 10% FBS, 1X mammary epithelial growth supplement (MEGS), 1% Penicillin–Streptomycin and 1% L-glutamine. The Postn-null cells were then infected using retroviral vectors encoding Postn (pMSCV-Postn) or the empty control vector (pMSCV) to be used as a re-expression model.

### Plasmids and transfections

Full-length Postn cDNA was amplified and cloned into the pMSCV retroviral vector. Infectious retroviruses were generated by transient transfections of retroviral vectors, a packaging plasmid (pUMCV; Addgene) and packaging vector (PCMV-VSV-G; Addgene) into 293 T cells (ATCC). Virus-containing medium was collected 24 h later, filtered through a 0.44-µm filter and then used for infection. The Postn gene was re-introduced into Postn −/− NeuNDL cells by infecting with pMSCV-Postn supernatants and 8 µg/ml of polybrene (Sigma). Control cells were infected with the empty pMSCV vector. Infected cells were then selected in 2 µg/ml of Puromycin (Sigma) for 5 days and subsequently maintained using 1 µg/ml of Puromycin. For siRNA transfections, 2 × 10^5^ cells were seeded and transfected with 400 nM of each siRNA in 1 mL of full medium for 24 h. The cells were transferred to MEGS-depleted medium for an additional 24 h and then assessed for Postn and Akt expression: Akt1 siRNA: AACGAUGGCACCUUUAUUGGC; Akt2 siRNA: AAACUCCUCGGCAAGGGCACC; Akt3 siRNA: AAGGAUGAAGUGGCACACACU.

### In vitro model and treatments

Cells were seeded in full growth medium (10% FBS, 1X MEGS DMEM:F12) for 24 h prior to replacing the medium. Briefly, the cells were washed and the medium was replenished using the components in 10% FBS DMEM:F12 as indicated in figure legends. Cells were treated using 3 ng/ml EGF (Sigma), 0.01 µg/ml IGF1 (Sigma), 0.5 µg/ml hydrocortisone (Sigma), 0.4% v/v BPE (Thermo Fisher Scientific) and TGFβ-1 recombinant protein (Thermo Fisher Scientific). The TGFβR inhibitor SB431542 (SelleckChem) was used at 5 µg/ml, 25 µM of PI3K inhibitor LY294002 (SelleckChem), 10 µM of AKT inhibitor MK-2206 (SelleckChem), 100 nM of FGFR inhibitor BGJ398 (SelleckChem) and 1 µg/ml PKC activator Phorbol 12-myristate 13-acetate (PMA; SelleckChem). The bFGF neutralizing antibody, clone bFM-1 (Millipore), was used at 2.5 µg/ml.

### Immunohistochemistry

Murine endpoint tumors and human TMA (Biomax BR962) were subjected to 10% formalin fixation and paraffin embedding, and the sections were processed as previously described [43]. Briefly, the sections were subjected to antigen retrieval using 10 mM citrate buffer (pH 6.0) in a pressure cooker. Sections were then quenched in 3% hydrogen peroxide, blocked and incubated overnight using the indicated primary antibody (Table 1) at 4 °C. Following incubation with secondary antibodies (Table 1), color development and detection was obtained using DAB substrate (Vector Laboratories). Counterstain was performed using hematoxylin. Sections were imaged using a Zeiss Axio Scan.Z1 slide scanner and then processed and analyzed using ImageJ. Quantification was done by assessing the amount of positive staining (brown pixels) using the color deconvolution tool (ImageJ) in the epithelial compartment of the tumors. A set threshold of 30% positive pixels was used to identify acquired versus non-acquired Postn expression status.

**Table 1:** List of antibodies

Antibody	Supplier	Catalog number
Donkey anti-Mouse IgG (H&L), HRP	Bio-Rad	170–6516
Goat anti-Rabbit IgG (H&L), HRP	Bio-Rad	170–6515
Rabbit monoclonal anti-phospho-AKT (Ser473)	Cell Signaling Technology	4060
Rabbit monoclonal anti-Phospho-Smad2 (Ser465/467)	Cell Signaling Technology	3108
Rabbit polyclonal anti-AKT	Cell Signaling Technology	9272
Rabbit monoclonal anti-Smad2 XP	Cell Signaling Technology	5339
Goat Polyclonal Mouse Periostin/OSF-2 Isoform 2 Antibody	R&D Systems	AF2955
Goat Polyclonal Human Periostin/OSF-2 Antibody	R&D Systems	AF3548
Mouse anti-Goat IgG-HRP	Santa Cruz Biotechnology	sc-2354
Mouse monoclonal Anti-p-ERK (Y204)	Santa Cruz Biotechnology	sc-7383
Mouse monoclonal anti-Smad4	Santa Cruz Biotechnology	sc-7966
Rabbit Polyclonal anti-ERK 2 Antibody	Santa Cruz Biotechnology	Sc-154
Mouse monoclonal anti-beta-Actin	Sigma-Aldrich	A5316
Donkey anti-Goat IgG Alexa Fluor 488	Thermo Fisher/Invitrogen	A-11055
Donkey anti-Rabbit IgG Alexa Fluor 594	Thermo Fisher/Invitrogen	A-21207

### Immunofluorescence

Cells were seeded, grown and treated on coverslips for the indicated time of the experiment. Cells were then washed in PBS, followed by fixation in 4% PFA for 10 min. Permeabilization was performed using 0.3% Triton-X for 5 min and blocked using 5% donkey serum in PBS for 30 min, followed by a 1-h incubation using the indicated primary antibody (Table 1) diluted in PBS containing 5% donkey serum. Coverslips were then incubated with Alexa Fluor secondary antibody (Table 1) for 1 h. Finally, the coverslips were washed and mounted using DAPI anti-fade (Invitrogen). Quantification of immunofluorescence was performed by manually counting of the total number of cells (DAPI) and the total Postn-positive cells or nuclear-positive cells for SMAD2. The proportion of positive cells was obtained by % = (# positive stain/total DAPI stain) × 100.

### Western blotting analysis

Tissues and cells were collected and homogenized in RIPA lysis buffer containing phosphatase and protease inhibitors as previously described [44]. The lysates were incubated at 4 °C with intermittent vortexing or multiple freeze/thaw cycles. The resulting lysates were then centrifuged at 12,000 rpm in a temperature-controlled centrifuge set to 4 °C for 10 min to clear the lysates of debris. Protein concentration were then determined using a Bio-Rad protein assay dye reagent. Equivalent amounts of proteins were incubated at 100 °C for 5 min in SDS sample buffer, followed by SDS-PAGE electrophoresis and transferred to a polyvinylidene difluoride (PVDF) membrane (Millipore). Membrane was then incubated with the indicated primary antibodies (Table 1) diluted in 5% BSA in TBS-T (0.1%). Membranes were then washed in TBS-T, and protein signals were detected following incubation with species-specific HRP-conjugated secondary antibodies (Table 1) and enhanced chemiluminescence reagent (PerkinElmer).

### RNA extraction and RT-PCR

Cell pellets were homogenized in Trizol reagent (Life Technologies), and total RNA was extracted using the manufacturer’s protocol. Total RNA was then subjected to on-column DNase treatment (QIAGEN) followed by first-strand cDNA synthesis using 500 ng of RNA, SuperScript III (Life Technologies), a mix of random primers (Invitrogen) and oligo dT (Invitrogen). Gene-specific primers and SYBR Green Reagent (Bio-Rad) were used to amplify the indicated targets. Reactions was carried out using an Applied Bioscience 7500 Fast-Real-Time PCR system. The forward and reverse primer sequences for Postn are 5′ AAGTTTGTTCGTGGCAGCAC 3′ and 5′ TTCTGTCACCGTTTCGCCTT 3′, respectively.

### Proliferation, migration and invasion assays

Cells were seeded at 50,000 cells per well in a 6-well plate, and a Vi-CELL XR cell viability analyzer was used to assess live cells using trypan blue every day for up to 4 days post-seeding. Three counts per well were performed with triplicate wells for each time point. Three biological replicates (*n* = 3) were performed for each cell line.

Transwell haptotaxis migration assays were performed by seeding cells in the top chamber of a collagen-coated Boyden Chamber insert (underside) in low serum conditions (1% FBS) with the same medium in the bottom chamber. Cells were left to migrate for 8 h; membranes were then fixed with 4% PFA and subjected to crystal violet (0.5%) staining. Manual counting was performed to assess the number of cells that successfully migrated through the pores of the membrane.

## Results

### Periostin expression is acquired in a subset of breast cancers independently of subtype

**Fig. 1:**
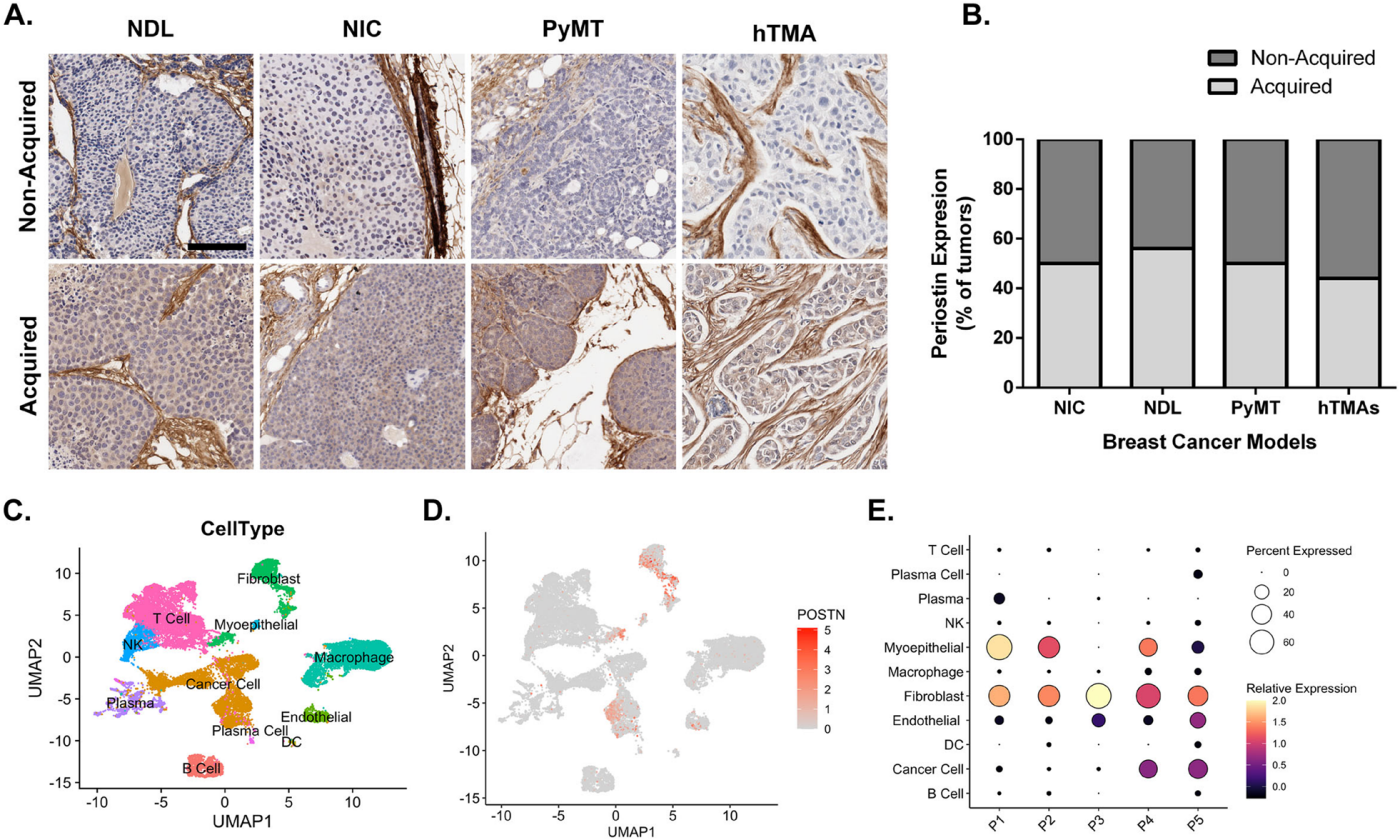
Epithelial Postn is acquired in a subset of breast cancers. **A** Immunohistochemistry for the expression of Postn was performed on mammary tumors from a panel of Neu-driven murine breast cancer models and on human tissue microarrays (TMAs). The models used were the MMTV-NeuNDL model (*n* = 9), MMTV-NIC (*n* = 10) and MMTV-PyMT (*n* = 8). The human TMA contained 72 cores with various levels of HER2 expression. Scale bar = 200 µm. **B** Tumors were classified as acquired or non-acquired according to their epithelial expression of Postn and were represented as a proportion of the total number in a bar graph. **C** Clustered UMAP embedding of scRNA-seq data of 24,271 cells from five triple-negative breast cancer tumors. Data and cell-type annotations were acquired from Wu et al*.* [1]. **D** Identical UMAP showing the distribution of *POSTN* expression across the cell types comprised in the tumor. Expression values correspond to log-transformed counts per 10 k transcripts.**)** Summary of the average *POSTN* expression (Z-score) in each cell type and the percentage of cells with *POSTN* detection for each patient (P1-5)

Postn has been shown to be expressed in the stroma of mammary tumors. However, the epithelial compartment has been found to express Postn in about 60% of cases and this has been correlated with poor prognosis [20]. To assess whether this pattern was also observed in murine tumors, we investigated the proportion of tumors that acquired epithelial expression of Postn in multiple HER2+ breast cancer murine models and compared it to human tissue microarrays (hTMA). Human breast cancer TMAs containing a mix of subtypes (Biomax BR962) and endpoint tumors from MMTV-NeuNDL [34], MMTV-NIC [35] and MMTV-PyMT [36] were subjected to immunohistochemistry using an anti-Postn antibody (Fig. 1A). Following staining, tumors that had more than 30% positive pixels in the epithelial compartments were classified as “acquired” Postn expression. After quantification, tumors and TMA cores were classified into a non-acquired (no/low POSTN epithelial expression) and acquired phenotype (high POSTN epithelial expression). As previously reported, acquired expression was observed in about 45% of human tumor cores, independently of the breast cancer subtype (Fig. 1B). Interestingly, murine tumors from all three HER2+ models showed acquired POSTN expression in approximately 50% of the tumors (Fig. 1B), suggesting that murine tumors acquire POSTN expression at a similar frequency. To further assess the *POSTN* expression pattern across the various cell types in breast tumors, we analyzed single-cell RNA sequencing data from five human TNBC tumors generated by Wu et al. [38]. Using these data, we assessed *POSTN* levels across cell types and found that its expression is largely specific to fibroblasts and cancer cell populations (Fig. 1C, D). Comparing its expression between individual tumors, *POSTN* is consistently expressed in tumor fibroblasts, but is expressed in cancer cells in only two of the five tumors (Fig. 1E), suggesting that stromal Postn expression is regulated differently than in epithelial cells. Combined with previous studies, these results show that Postn expression in the epithelial compartment is acquired in about 40–50% of breast cancers irrespective of subtypes.

### Mammary epithelial growth supplement represses Postn expression in vitro

**Fig. 2:**
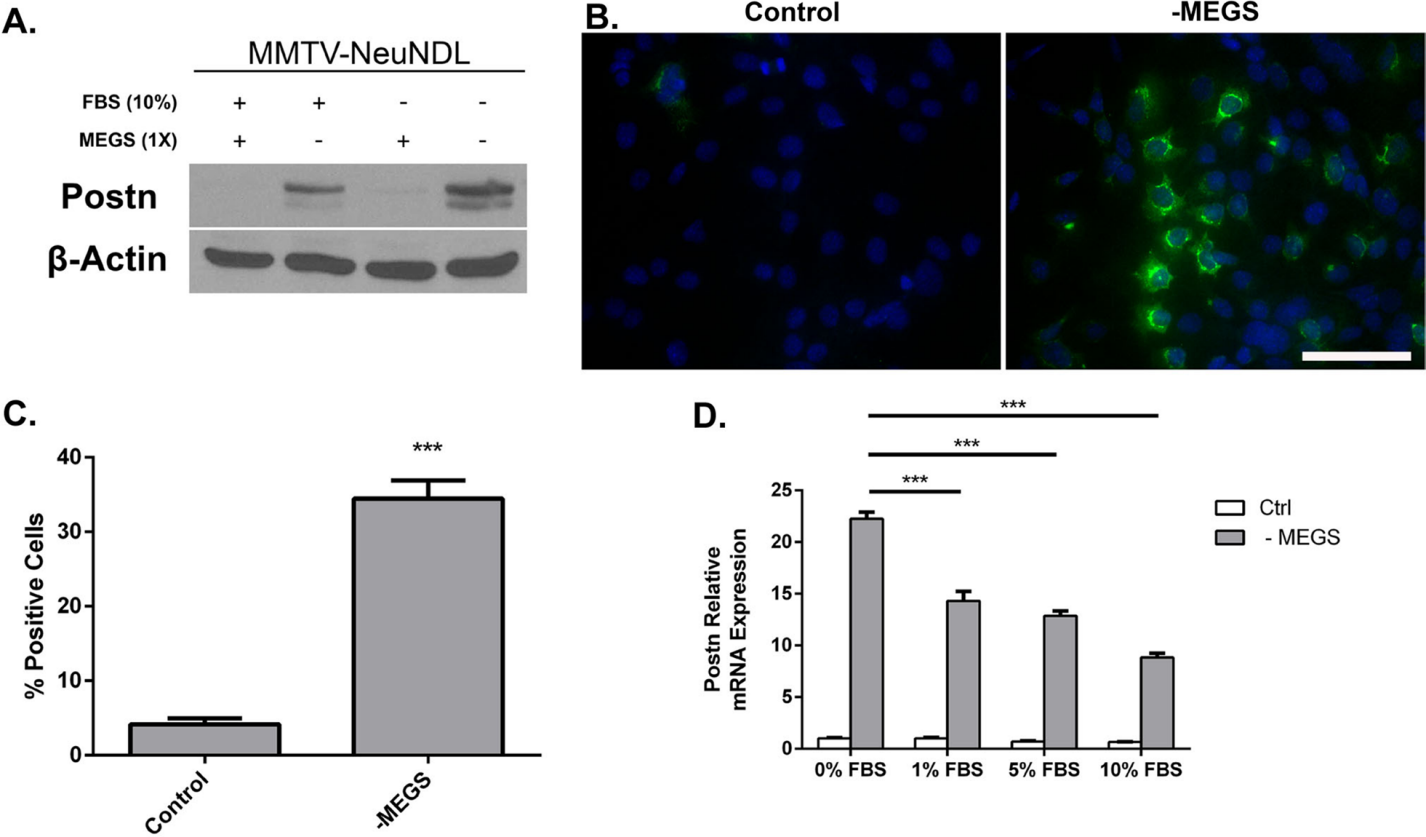
Mammary epithelial growth supplement represses Postn expression in NeuNDL cells. **A** NeuNDL cells were grown in the presence or absence of FBS and MEGS as well as with neither and a combination of both for 24 h. Postn expression was assessed by western blotting analysis with β-actin as a loading control. **B** NeuNDL cells were seeded on coverslips, grown in the presence or absence of MEGS for 48 h and immunostained. Individual coverslips were stained for DAPI and POSTN. Scale bar = 100 µm **C** Representative images were counted to assess the percentage of POSTN expressing cells in both conditions. Data are represented as mean ± SEM. *N* = 10, ***P ≤ 0.001. **D** NeuNDL cells were treated with or without MEGS in varying FBS concentration from 0 to 10%, and Postn mRNA expression was assessed 24 h post-treatment. Data are represented as mean ± SEM. *N* = 3, ***P ≤ 0.001

**Fig. 3:**
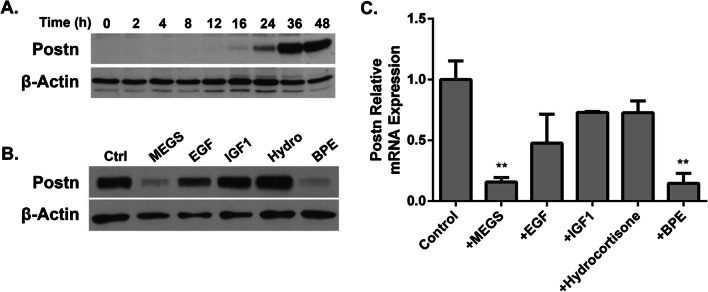
Bovine pituitary extract is the active component repressing Postn expression. **A** NeuNDL cells were seeded and grown in MEGS-depleted medium for up to 48 h, and POSTN expression was assessed at multiple time points. β-Actin was used as a loading control. **B** NeuNDL cells were seeded and then grown in either MEGS, or individual components of MEGS (EGF 3 ng/ml, IFG1 0.01 µg/ml, hydrocortisone 0.5 µg/ml, BPE 0.4% v/v), and Postn protein levels and mRNA expression **C** were assessed after 24 h. mRNA expression data are represented as mean ± SEM. *N* = 3, **P ≤ 0.01

To investigate the role of Postn in tumor cell growth and invasion in vitro, we isolated wild-type and Postn-deficient tumor cells from MMTV-NeuNDL and MMTV-NeuNDL × Postn-null animals, respectively. To minimize the effect of cell line variability, Postn was re-expressed in a Postn-deficient line and stable pools were compared. Interestingly, the loss of Postn did not show any differences in growth rate (Additional file 1: Figure S1) or migration (Additional file 2: Figure S2A, B) in vitro. Similar studies using basement membrane extracts also showed no difference in invasive potential (not shown). One possibility is that the effects of Postn on tumor cells are mediated through reorganization of the microenvironment that would not occur in culture. Serendipitously, we observed that prolonged culture or starvation through mammary epithelial growth supplement (MEGS) removal induced Postn expression in cultured tumor cells (Additional file 3: Figure S3A). Interestingly, this induction was also more prominent under low serum conditions (Fig. 2A). A MMTV-NeuNDL isolate showing strong Postn induction at 36 h was selected for the remainder of the studies. To assess Postn expression at the single-cell level, we performed Postn immunofluorescence on cells growing in full medium or MEGS-depleted conditions for 24 h (Fig. 2B). Quantification of Postn expressing cells from representative images showed a significant increase in the proportion of cells expressing Postn in MEGS-depleted medium (Fig. 2C). To assess whether Postn induction was due to increased mRNA levels, we performed Q-PCR analysis on MEGS-depleted cultures. Postn mRNA levels were monitored in MEGS-depleted medium with varying FBS concentrations from 0 to 10%. As shown in Fig. 2D, MEGS depletion resulted in an increase in *Postn* mRNA that was also suppressed at high serum concentrations. Together, our data show that components in the MEGS and serum can mediate the repressive effect on Postn gene expression. To further investigate the kinetics and mechanisms of MEGS-mediated repression, we performed time course studies following MEGS removal. Western blotting analysis shows that Postn expression is induced 16 h following MEGS removal and reaches maximal levels at about 36 h (Fig. 3A). The MEGS supplement contains a mixture of growth factors (EGF, IFG1, Hydrocortisone, BPE), and repressive ability of each individual component was assessed at 24 h. Western blot analysis shows that, within the MEGS, only the bovine pituitary extract (BPE) demonstrates strong repressive ability (Fig. 3B)**.** Similarly, Q-PCR analysis shows that the BPE is necessary and sufficient to inhibit Postn mRNA induction (Fig. 3C). Additional analyses showed that the repressive component(s) responsible for Postn repression found in BPE are heat labile (Additional file 3: Figure S3B) and that they are acting in a dose-dependent manner (Additional file 3: Figure S3C). Albeit with different kinetics, the induction of Postn following MEGS depletion was also observed in different isolates from MMTV-NeuNDL and MMTV-NIC tumors, suggesting that it is not cell line specific (Additional file 4: Figure S4A, B). These data suggest that one or more components of the BPE confer the repressive activity on Postn gene expression.

### Modulation of Postn expression by TGFβ and bFGF is SMAD2-independent

**Fig. 4:**
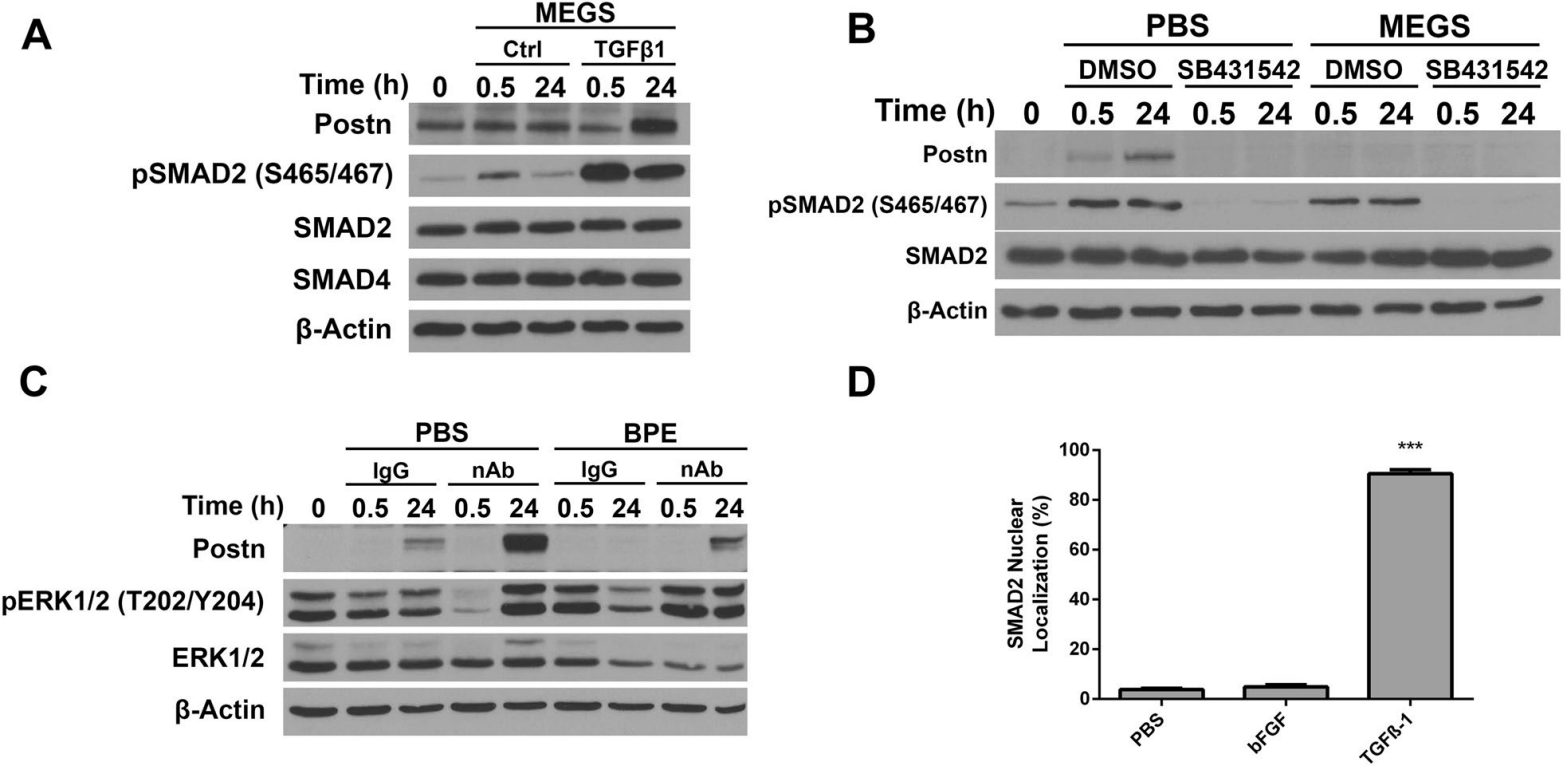
Modulation of Postn expression by FGFR and TGFβ signaling. **A** NeuNDL cells were treated in the presence of MEGS with or without 10 ng/ml of TGFβ-1 for 0.5 and 24 h and assessed for Postn protein levels. pSMAD2 (S465/467) was used as a control for the activation of the TGFβR. **B** Postn protein levels monitored in NeuNDL cells were treated with 5 µg/ml TGFβR inhibitor SB431542 or DMSO in either MEGS-depleted (PBS) or MEGS-supplemented medium for 0.5 and 24 h. pSMAD2 (S465/467) was used as a control for TFGβR inhibition. **C** MEGS-depleted (PBS) and BPE-supplemented media were pretreated with bFGF neutralizing antibody (nAb) or control IgG for 1 h at 4 °C prior to addition to the NeuNDL cultures and western blotting for Postn. **D** Immunofluorescence for SMAD2 nuclear localization was performed following a 24-h treatment with PBS, 10 ng/ml bFGF or 10 ng/ml TGFβ-1 of MEGS-depleted cultures. Manual counting of representative images was conducted to assess localization. Data are represented as mean ± SEM. *N* = 10, ***P ≤ 0.001 (**D**)

Several studies have implicated hormones and growth factors in the regulation of Postn expression [45]. Based on these findings, we tested the repressive ability of potential pituitary hormones such as prolactin, luteinizing hormone and follicle-stimulating hormone. None of the hormones tested, including cAMP analogs, showed any effect on Postn gene expression (not shown). Interestingly, Postn has been shown to be induced by TGFβ and bFGF [8, 19], both produced by the pituitary [46, 47]. Therefore, we tested their effect on Postn expression upon MEGS removal. Western blotting analysis confirmed the induction of Postn in these cells following a 24-h TGFβ-1 treatment (Fig. 4A). Treatment with the TGFβR inhibitor SB431542 on MEGS-depleted cultures prevented Postn induction (Fig. 4B)**.** The decrease in phospho-SMAD2 levels confirmed the activity of the inhibitor (Fig. 4B).

We next assessed the effect of bFGF on Postn expression. Cultures were treated with bFGF in the presence or absence of MEGS. Surprisingly, bFGF treatment in the MEGS-depleted cultures repressed Postn induction (Additional file 5: Figure S5). This was partially reversed upon treatment with the pan-FGFR inhibitor (BGJ398; Additional file 5: Figure S5), suggesting that bFGF-mediated Postn repression proceeds through FGFR signaling. Further supporting the role of bFGF in BPE-mediated repression, treatment of the cultures with BPE that has been pre-incubated with an anti-bFGF neutralizing antibody is able to partially restore Postn induction (Fig. 4C). Additionally, cells treated with TGFβ-1 in combination with bFGF showed a repression of Postn, suggesting that FGFR signaling activates a repressive cross talk to the TGFβ pathway in this context (Additional file 6: Figure S6). Supporting the presence of FGF2 in BPE, a protein-based, thermolabile and growth-promoting “Fibroblast Growth Factor” (FGF2) activity was also identified in extracts from bovine pituitary glands decades ago [48–51].

To test whether the activation of Postn following MEGS removal proceeded through TGFβ canonical signaling (SMAD-dependent), we assessed nuclear localization of SMAD2 protein as a readout for SMAD complex activation. Although it induces Postn, MEGS removal did not alter SMAD2 nuclear import (Fig. 4D and Additional file 7: Figure S7). Similarly, bFGF treatment did not affect SMAD2 distribution. These results suggest that MEGS-mediated repression of Postn expression occurs through a bFGF-dependent mechanism and that Postn induction through MEGS removal is SMAD-independent.

### PI3K/AKT pathway activity is required for the induction of Postn

**Fig. 5:**
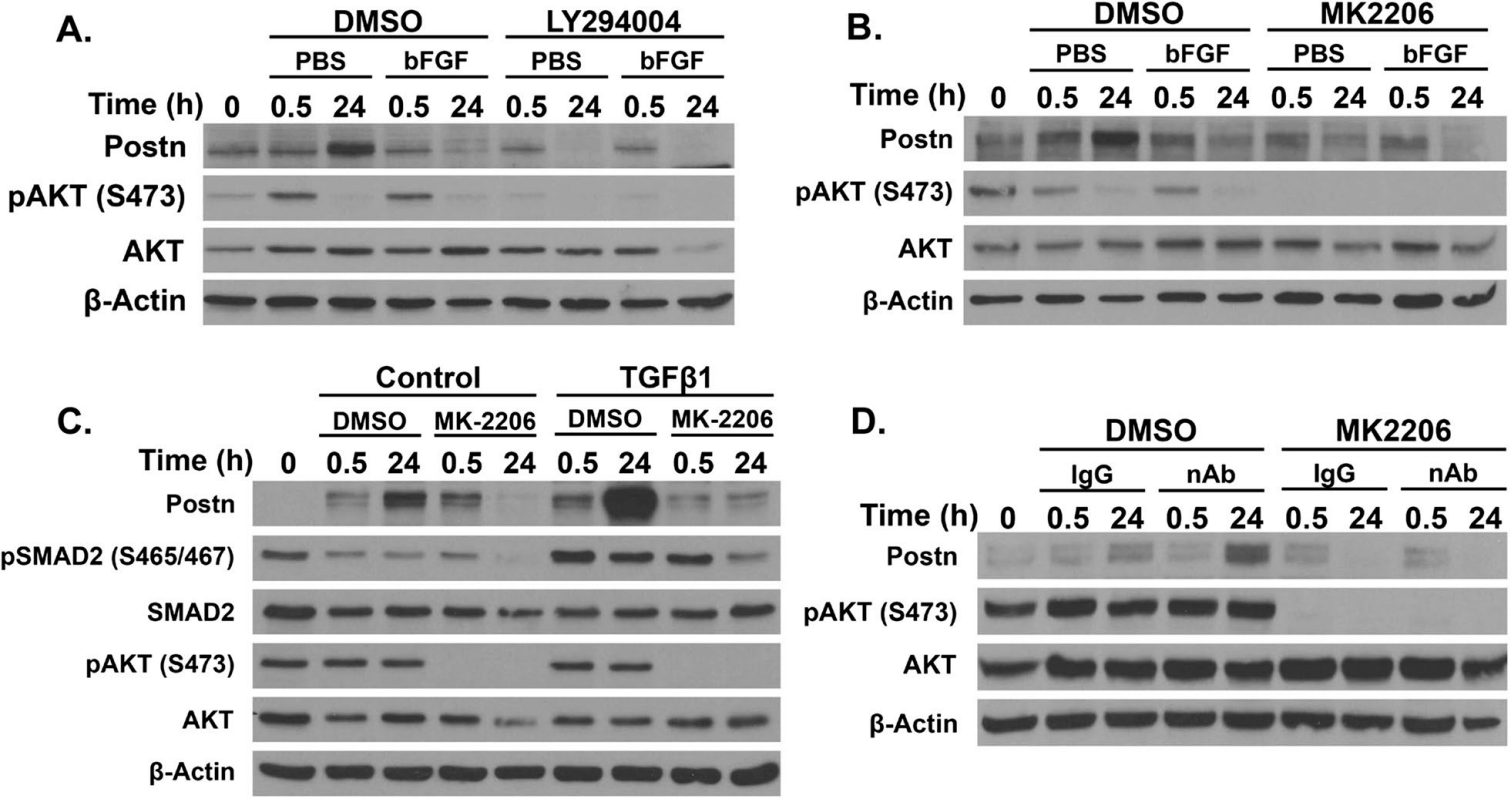
PI3K/AKT pathway activity is required for the induction of Postn by bFGF removal and TGFβ activation. **A** NeuNDL cells were treated with 25 µM LY294004 or DMSO control in both PBS and 10 ng/ml bFGF supplemented medium in the absence of MEGS for 0.5 and 24 h. pAKT (S473) was used as a control for the inhibition of PI3K. **B** NeuNDL cells were treated the exact same way as in (A) but with 10 µM of MK-2206 instead of LY294004. **C** NeuNDL cells were treated with or without 10 ng/ml TGFβ-1 in combination with either 10 µM MK-2206 or DMSO controls. pSMAD2 (S465/467) and pAKT (S473) were used as control for TGFβR activation and AKT inhibition, respectively. **D** Medium was pretreated with bFGF neutralizing antibody or control IgG in combination with either DMSO or 10 µM MK-2206. pAKT (S473) was used as control for AKT inhibition. In all panel PBS designates MEGS-depleted conditions

**Fig. 6:**
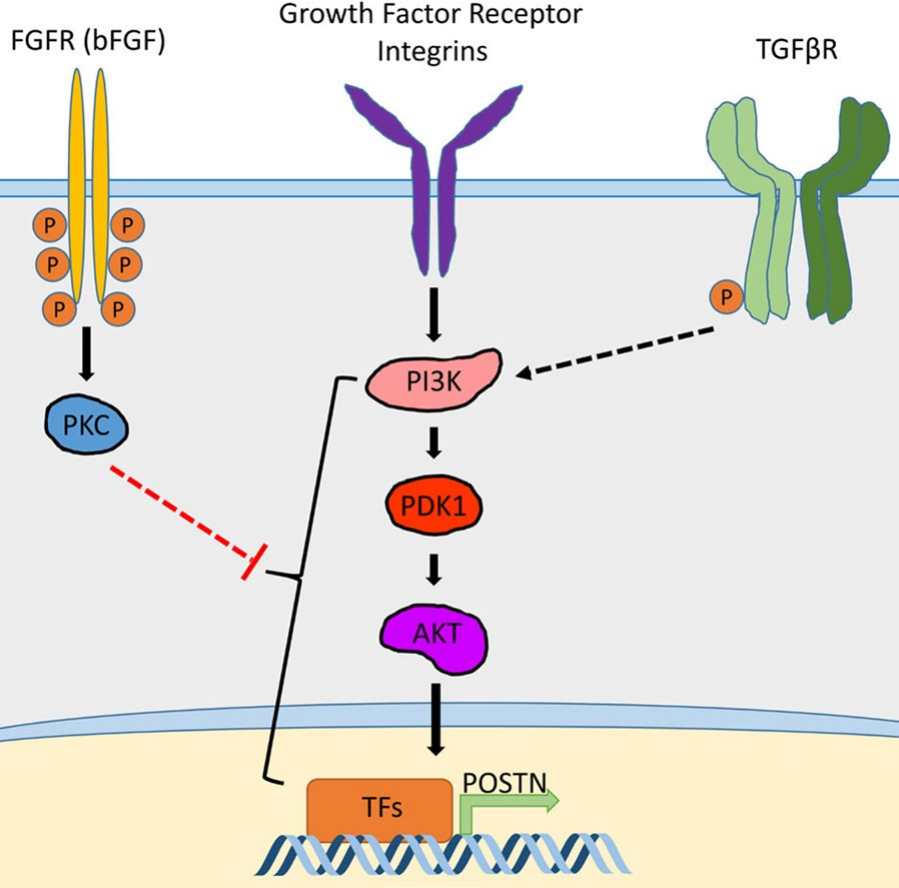
Postn gene expression is regulated by both bFGF and TGFβ. Schematic summarizing the regulation of Postn by TGFβ and FGF2. PI3K/AKT is the main drive of Postn expression. Although the exact effectors remain to be identified, FGFR signaling can repress Postn expression by acting on the PI3K/AKT pathway at one or multiple levels. TGFβ signaling is shown to activate PI3K to promote AKT activation and the expression of Postn. Other growth factor receptors and surface integrins shown to activate Postn through AKT are also represented

Both the TGFβR and FGFR pathways signal to multiple systems (reviewed in [52, 53]). To further dissect the mechanisms of Postn gene regulation, we treated the cultures with several inhibitors in the presence or absence of MEGS. Combined with MEGS removal, treatment of the cells with inhibitors for p38 (SB203580) or MEK1 (U0126) did not affect Postn expression, suggesting that their downstream signals do not mediate induction or repression (not shown). Similarly, MEGS removal did not affect phospho-c-Jun levels, suggesting that JNK activity is not critical for Postn upregulation (not shown). We then assessed the role of the PI3K pathway, activated by both TGFβ and FGFR and an important pro-tumorigenic signal in breast cancer. Treatment of MEGS-depleted cultures with the PI3K inhibitor LY294004 or the pan-Akt inhibitor MK-2206 resulted in a decrease in pAkt-S473 levels by 24 h and a complete block of Postn induction (Fig. 5A, B). However, bFGF treatment in the presence of the same inhibitors had no effect on the repressive ability of bFGF (Fig. 5A, B). Furthermore, western blotting analysis of cells subjected to TGFβ-1 treatment in combination with MK-2206 did not induce Postn expression (Fig. 5C). Similarly, bFGF neutralizing antibody treatment in the absence of MEGS resulted in Postn repression in the presence of MK-2206 (Fig. 5D). As for MK-2206, knockdown of Akt1 markedly reduced Postn induction following MEGS removal (Additional file 8: Figure S8). Interestingly, Akt2 or Akt3 knockdowns had no effect (not shown). Supporting a role for FGFR downstream signaling in Postn repression, treatment with the protein kinase C (PKC) activator PMA was as efficient as bFGF alone in preventing Postn induction, suggesting that PKC plays a role in Postn repression (Additional file 9: Figure S9). Together, these data suggest that Postn induction requires the PI3K/Akt pathway that can be repressed by bFGF signaling, partly through PKC (Fig. 6).

## Discussion

Our data show that Postn regulation in breast cancer cells is quite complex, involving multiple signaling pathways. The control of its expression can be achieved by the FGFR, TGFβ and PI3K/AKT pathways. Our data show that the removal of bFGF from the culture medium is sufficient to induce Postn gene expression. This requires TGFβ/PI3K/Akt signaling. Interestingly, upregulation of Postn by this pathway was found to be SMAD-independent. In addition, Postn repression through FGFR activation appears to be PKC-dependent. Overall, our data suggest that PI3K/AKT is the major pathway responsible for the regulation of Postn in cultured breast cancer tumor cells.

Postn has been identified as an indicator of poor prognosis whether it is found in the serum of patients or expressed by the tumor cells. Previous observations showed that Postn from epithelial and stromal origin (CAFs) might have distinct but important role in promoting tumorigenesis [33, 54]. TGFβ has been shown to have tumor suppressor activity by inducing cell cycle arrest and apoptosis [55]. Although, in the context of transformation, it can promote EMT which facilitates cancer cell migration and metastasis [56], it is unclear how this switch from tumor suppressor to promoter of metastasis occurs. FGF2 downregulation has been identified as a marker of poor prognosis in breast cancer using a methylation-based bioinformatic approach [57]. The PI3K/AKT pathway is generally associated with cancer progression. The PIK3CA gene, coding for a subunit of the catalytic domain of PI3K, is often mutated or amplified in breast cancer, and therapeutic [58] inhibition of this pathway has shown some promising results in some types of breast cancer (Reviewed in [59]). Interestingly, our data show that Postn gene expression also appears to proceed through a TGFβ/AKT pathway in this context. Together with the loss of FGF2 expression [57], activation of the TGFβ/AKT axis could lead to Postn induction in tumor cells, contributing to increased invasion and worse outcomes.

In an attempt to identify transcription factors and consensus transcription factor binding sites, we have analyzed DNA fragments up to 10 kb upstream of the Postn start site using luciferase reporter assays. No significant change in reporter activity was observed following MEGS removal system (not shown). This suggests that the critical elements regulating Postn in our model are likely to be located further upstream or downstream of the start site. A study used a similar approach and identified a YingYang-1 (YY1) consensus binding sequence in the − 4 kb proximal promotor region validating this as a regulator of Postn in Schwann cells. Interestingly, no other cell types responded to that transcription factor [60], suggesting a cell-type specificity and likely a context-dependent regulation of Postn. Supporting this, another study identified recombination signal binding protein for immunoglobulin kappa J region (RBPJ) binding to a region relatively close to the YY1 binding sites, specifically in hepatocytes and liver cancer cells [61].

Postn has been previously identified as a target gene of TGFβ signaling in osteoblasts [8]. TGFβ signaling has also been shown to have cross talk to the PI3K/AKT pathways [62, 63]. Interestingly, binding and activation of p85, the regulatory subunit of PI3K, by TGFβRI and TGFβRII, have been demonstrated in mammary epithelial cells [64]. Whether this is the case in MEGS-depleted cells remains to be tested. Nevertheless, a cross talk between activation of TGFβ signaling and PI3K/AKT pathway activation has been well documented [65, 66]. Similarly, in cancer models, PI3K/AKT signaling has been shown to antagonize TGFβ-induced apoptosis and growth arrest by interacting directly with SMAD3 [67, 68]. It has been proposed that activation of PI3K/AKT leads to TGF-β-dependent pro-oncogenic responses in breast cancer [69].

In contrast to our observations, a study using the non-small-cell lung cancer cell line A549 showed that bFGF could induce Postn expression in a PI3K/AKT-dependent manner [19]. It is likely that Postn expression is subject to tissue- as well as tumor-specific regulation. However, it is interesting to note that Postn induction proceeds through PI3K/AKT in both models. Interestingly, germline and conditional bFGF (FGF2) loss of function studies showed some phenotypic overlap with Postn loss of function studies. Basic FGF-null and Postn-null phenotypes both include muscle and skeletal development issues [37, 70, 71]. Similarly, the conditional deletion of FGF2 results in decreased cardiac hypertrophy induced by ischemic injury, delayed wound healing and increased bone mineralization, while Postn has been linked to cardiac issues, wound healing and bone regeneration [72–76].

Although it is not clear what the downstream AKT effectors are, the signaling mechanisms identified here demonstrate interplay between FGFR, TGFβ and PI3K/AKT pathways. We have shown that blocking the TGFβ or PI3K/AKT pathway leads to a complete repression of Postn, while bFGF is also repressing its expression. Our data suggest that bFGF signaling, through PKC, is repressing the PI3K/AKT pathway. One possibility is that it prevents TGFβR from recruiting and signaling to PI3K. Alternatively, the FGF signal prevents AKT signaling to its downstream effectors or components of the transcriptional complex. FGFR signaling might also be competing with the TGFβ/PI3K/AKT axis. This could potentially imply that FGFR signaling promotes a more proliferative state, while the TGFβ/PI3K/AKT axis could promote a more mesenchymal and invasive state by inducing Postn expression. Supporting this, TGFβ has been shown to activate PI3K to promote EMT [77]. A study showed that PKCα can suppress AKT activity through a protein phosphatase 2A (PP2A)-dependent mechanism [78]. Given the number of PKC isoforms and targets, it is also possible that some of its substrates are acting throughout the TGFβ/PI3K/AKT axis (Reviewed in [79]). For instance, the SKI protein, negatively regulating TGFβ through the SMAD family, is a potential PKC substrate [80].

## Conclusions

Taken together, our studies have identified the TGFβ/PI3K/AKT and FGFR as major pathways regulating Postn expression in HER2+ breast cancer cells. These have complex regulation and cross talk mechanisms that are likely to be cell type and cancer specific. The identification of critical transcriptional elements and additional FGF-TGFβ cross talk pathways will help delineate the mechanisms responsible for acquired Postn expression in breast cancer cells.

## Supplementary Information

**Additional file 1: Figure S1**. Growth rate is not affected in Postn-null breast cancer cells. NeuNDL Postn −/− cells were infected with retroviruses encoding Postn (pMSCV-Postn) or the empty vector (pMSCV). The cells were seeded and assessed for proliferation for up to 4-day post-seeding. This was performed on 2 independent isolates of Postn −/− cells (A and B). Data is represented as mean ± SEM. *N* = 3.

**Additional file 2: Figure S2**. Migration rate is unaffected by forced expression of Postn. (A) Haptotaxis was assessed between NeuNDL Postn −/− cells stably re-expressing Postn (pMSCV-Postn) or the empty vector control (pMSCV). This was performed on 2 independent isolates of Postn −/− cells (A and B). Representative pictures of crystal violet stained membranes are shown. Scale bar = 200 µm. (B) Quantification by manual counting of 10 representative field of view per membrane is shown in the bar graph. Data is represented as mean ± SEM. *N* = 3.

**Additional file 3: Figure S3**. A short-lived heat labile BPE component represses Postn expression. (A) NeuNDL cells were left to grow in MEGS supplemented media and assessed for Postn protein expression after 2, 4 and 6 days without changing the medium. Upregulation of Postn suggests the depletion of a repressive activity. (B) NeuNDL cells were treated with PBS, MEGS and BPE were pre-incubated at 37°C or 100°C for 15 min to assess the heat sensitivity of the repressive components. Postn relative mRNA expression is shown in the bar graph for each condition. (C) NeuNDL cells were treated using 0%, 0.1%, 0.2% and 0.4% BPE to assess a dose-dependent relationship for the repression of Postn. Postn relative mRNA expression is shown in the bar graph for each condition. Data is represented as mean ± SEM. * = *P* ≤ 0.05 (C)

**Additional file 4: Figure S4**. Removal of MEGS also induces Postn in other cell lines. An independent isolate (iso 2) of a MMTV-NeuNDL tumor (A) and an isolate from a MMTV-NIC tumor (B) were subjected to MEGS-deficient medium and Postn protein expression was assessed up to 96 h post-plating.

**Additional file 5: Figure S5**. FGF2-mediated repression of Postn is rescued by an FGFR inhibitor. NeuNDL cells were plated in the absence of MEGS with 10ng/ml bFGF in combination with a pan-FGFR inhibitor BGJ398 at 100 nM. Postn protein expression was assessed by western blotting at 0.5- and 24-h post-treatment.

**Additional file 6: Figure S6**. TGFβ-mediated induction of Postn is blocked by bFGF. NeuNDL cells were plated in the absence of MEGS and treated with 10 ng/ml of TGFβ-1 in combination with 10ng/ml of bFGF. Periostin protein expression was assessed 0.5- and 24-h post-treatment by western blotting analysis. Phospho-specific SMAD2 was used as a control for the activation of TGFβRs.

**Additional file 7: Figure S7**. Postn induction is independent of SMAD2 activation. NeuNDL cells were subjected to MEGS-depleted conditions supplemented with either PBS, bFGF (10 ng/ml) or TGFβ-1 (10 ng/ml). Immunofluorescence using an anti-SMAD2 antibody is shown as a single channel and merged with DAPI. MEGS removal or FGF addition did not affect SMAD2 localization. Scale bar = 100 µm

**Additional file 8: Figure S8**. Akt1 knock down impairs Postn induction following MEGS depletion. Cultures were transfected with an Akt1 siRNA (or non-targeting control; NTC) in the presence of MEGS and then switched to MEGS depleted medium for 24 h. Postn and Akt1 expression were assessed by western blot.

**Additional file 9: Figure S9**. PKC activation can repress Postn expression and enhanced the effect of bFGF. NeuNDL cells were treated in absence of MEGS with PMA (1 µg/ml) in combination with bFGF (10 ng/ml). Postn expression was assessed by western blotting analysis and phospho-ERK1/2 was used as a control for PKC activation by PMA.

## Data Availability

The datasets analyzed during the current study were generated by Wu et al. [38]*.* Raw UMI counts and cell metadata were acquired from ENA accession PRJEB35405.

## Abbreviations

BMP-1: Bone morphogenetic protein 1
EMT: Epithelial-to-mesenchymal transition
ESR1: Estrogen receptor 1
FAK: Focal adhesion kinase
FGF: Fibroblast growth factor
HER2: Human epidermal growth factor receptor 2
LOX: Lysyl oxidase
MEGS: Mammary epithelial growth supplement
OSF-2: Osteoblast-specific factor 2
PI3K: Phosphoinositide 3-kinase
PGR: Progesterone receptor
TMA: Tissue microarray

## References

[1] Bray F, Ferlay J, Soerjomataram I, Siegel RL, Torre LA, Jemal A (2018). Global cancer statistics 2018: GLOBOCAN estimates of incidence and mortality worldwide for 36 cancers in 185 countries. CA Cancer J Clin.

[2] Curtis C, Shah SP, Chin SF, Turashvili G, Rueda OM, Dunning MJ (2012). The genomic and transcriptomic architecture of 2,000 breast tumours reveals novel subgroups. Nature.

[3] Perou CM, Sorlie T, Eisen MB, van de Rijn M, Jeffrey SS, Rees CA (2000). Molecular portraits of human breast tumours. Nature.

[4] Slamon DJ, Godolphin W, Jones LA, Holt JA, Wong SG, Keith DE (1989). Studies of the HER-2/neu proto-oncogene in human breast and ovarian cancer. Science.

[5] Takeshita S, Kikuno R, Tezuka K, Amann E (1993). Osteoblast-specific factor 2: cloning of a putative bone adhesion protein with homology with the insect protein fasciclin I. Biochem J.

[6] Kii I, Nishiyama T, Li M, Matsumoto K, Saito M, Amizuka N (2010). Incorporation of tenascin-C into the extracellular matrix by periostin underlies an extracellular meshwork architecture. J Biol Chem.

[7] Maruhashi T, Kii I, Saito M, Kudo A (2010). Interaction between periostin and BMP-1 promotes proteolytic activation of lysyl oxidase. J Biol Chem.

[8] Horiuchi K, Amizuka N, Takeshita S, Takamatsu H, Katsuura M, Ozawa H (1999). Identification and characterization of a novel protein, periostin, with restricted expression to periosteum and periodontal ligament and increased expression by transforming growth factor beta. J Bone Miner Res.

[9] Bao S, Ouyang G, Bai X, Huang Z, Ma C, Liu M (2004). Periostin potently promotes metastatic growth of colon cancer by augmenting cell survival via the Akt/PKB pathway. Cancer Cell.

[10] Li G, Jin R, Norris RA, Zhang L, Yu S, Wu F (2010). Periostin mediates vascular smooth muscle cell migration through the integrins alphavbeta3 and alphavbeta5 and focal adhesion kinase (FAK) pathway. Atherosclerosis.

[11] Baril P, Gangeswaran R, Mahon PC, Caulee K, Kocher HM, Harada T (2007). Periostin promotes invasiveness and resistance of pancreatic cancer cells to hypoxia-induced cell death: role of the beta4 integrin and the PI3k pathway. Oncogene.

[12] Christiansen DL, Huang EK, Silver FH (2000). Assembly of type I collagen: fusion of fibril subunits and the influence of fibril diameter on mechanical properties. Matrix Biol.

[13] Norris RA, Damon B, Mironov V, Kasyanov V, Ramamurthi A, Moreno-Rodriguez R (2007). Periostin regulates collagen fibrillogenesis and the biomechanical properties of connective tissues. J Cell Biochem.

[14] Oshima A, Tanabe H, Yan T, Lowe GN, Glackin CA, Kudo A (2002). A novel mechanism for the regulation of osteoblast differentiation: transcription of periostin, a member of the fasciclin I family, is regulated by the bHLH transcription factor, twist. J Cell Biochem.

[15] Li G, Oparil S, Sanders JM, Zhang L, Dai M, Chen LB (2006). Phosphatidylinositol-3-kinase signaling mediates vascular smooth muscle cell expression of periostin in vivo and in vitro. Atherosclerosis.

[16] Takayama G, Arima K, Kanaji T, Toda S, Tanaka H, Shoji S (2006). Periostin: a novel component of subepithelial fibrosis of bronchial asthma downstream of IL-4 and IL-13 signals. J Allergy Clin Immunol.

[17] Lee MJ, Heo SC, Shin SH, Kwon YW, Do EK, Suh DS (2013). Oncostatin M promotes mesenchymal stem cell-stimulated tumor growth through a paracrine mechanism involving periostin and TGFBI. Int J Biochem Cell Biol.

[18] Rani S, Barbe MF, Barr AE, Litivn J (2010). Role of TNF alpha and PLF in bone remodeling in a rat model of repetitive reaching and grasping. J Cell Physiol.

[19] Ouyang G, Liu M, Ruan K, Song G, Mao Y, Bao S (2009). Upregulated expression of periostin by hypoxia in non-small-cell lung cancer cells promotes cell survival via the Akt/PKB pathway. Cancer Lett.

[20] Kim GE, Lee JS, Park MH, Yoon JH (2017). Epithelial periostin expression is correlated with poor survival in patients with invasive breast carcinoma. PLoS ONE.

[21] Rachner TD, Göbel A, Hoffmann O, Erdmann K, Kasimir-Bauer S, Breining D (2020). High serum levels of periostin are associated with a poor survival in breast cancer. Breast Cancer Res Treat.

[22] Lee YJ, Kim IS, Park SA, Kim Y, Lee JE, Noh DY (2013). Periostin-binding DNA aptamer inhibits breast cancer growth and metastasis. Mol Ther.

[23] Malanchi I, Santamaria-Martinez A, Susanto E, Peng H, Lehr HA, Delaloye JF (2012). Interactions between cancer stem cells and their niche govern metastatic colonization. Nature.

[24] Shao R, Bao S, Bai X, Blanchette C, Anderson RM, Dang T (2004). Acquired expression of periostin by human breast cancers promotes tumor angiogenesis through up-regulation of vascular endothelial growth factor receptor 2 expression. Mol Cell Biol.

[25] Nuzzo PV, Rubagotti A, Zinoli L, Salvi S, Boccardo S, Boccardo F (2016). The prognostic value of stromal and epithelial periostin expression in human breast cancer: correlation with clinical pathological features and mortality outcome. BMC Cancer.

[26] Powers CJ, McLeskey SW, Wellstein A (2000). Fibroblast growth factors, their receptors and signaling. Endocr Relat Cancer.

[27] Akl MR, Nagpal P, Ayoub NM, Tai B, Prabhu SA, Capac CM (2016). Molecular and clinical significance of fibroblast growth factor 2 (FGF2 /bFGF) in malignancies of solid and hematological cancers for personalized therapies. Oncotarget.

[28] Ibrahimi OA, Zhang F, Hrstka SC, Mohammadi M, Linhardt RJ (2004). Kinetic model for FGF, FGFR, and proteoglycan signal transduction complex assembly. Biochemistry.

[29] Wang ZG, Wang Y, Huang Y, Lu Q, Zheng L, Hu D (2015). bFGF regulates autophagy and ubiquitinated protein accumulation induced by myocardial ischemia/reperfusion via the activation of the PI3K/Akt/mTOR pathway. Sci Rep.

[30] Wang P, Li J, Zhang C, Luo L, Ni S, Tang Z (2019). bFGF overexpression adipose derived mesenchymal stem cells improved the survival of pulmonary arterial endothelial cells via PI3k/Akt signaling pathway. Int J Biochem Cell Biol.

[31] Krejci P, Faitova J, Laurell H, Hampl A, Dvorak P (2003). FGF-2 expression and its action in human leukemia and lymphoma cell lines. Leukemia.

[32] Gleave M, Hsieh JT, Gao CA, von Eschenbach AC, Chung LW (1991). Acceleration of human prostate cancer growth in vivo by factors produced by prostate and bone fibroblasts. Cancer Res.

[33] Ratajczak-Wielgomas K, Grzegrzolka J, Piotrowska A, Matkowski R, Wojnar A, Rys J (2017). Expression of periostin in breast cancer cells. Int J Oncol.

[34] Siegel PM, Dankort DL, Hardy WR, Muller WJ (1994). Novel activating mutations in the neu proto-oncogene involved in induction of mammary tumors. Mol Cell Biol.

[35] Ursini-Siegel J, Hardy WR, Zuo D, Lam SH, Sanguin-Gendreau V, Cardiff RD (2008). ShcA signalling is essential for tumour progression in mouse models of human breast cancer. EMBO J.

[36] Guy CT, Cardiff RD, Muller WJ (1992). Induction of mammary tumors by expression of polyomavirus middle T oncogene: a transgenic mouse model for metastatic disease. Mol Cell Biol.

[37] Rios H, Koushik SV, Wang H, Wang J, Zhou HM, Lindsley A (2005). Periostin null mice exhibit dwarfism, incisor enamel defects, and an early-onset periodontal disease-like phenotype. Mol Cell Biol.

[38] Wu SZ, Roden DL, Wang C, Holliday H, Harvey K, Cazet AS (2020). Stromal cell diversity associated with immune evasion in human triple-negative breast cancer. EMBO J.

[39] Hao Y, Hao S, Andersen-Nissen E, Mauck WM, Zheng S, Butler A (2020). Integrated analysis of multimodal single-cell data. Cell.

[40] Hafemeister C, Satija R (2019). Normalization and variance stabilization of single-cell RNA-seq data using regularized negative binomial regression. Genome Biol.

[41] Schubert M, Klinger B, Klünemann M, Sieber A, Uhlitz F, Sauer S (2018). Perturbation-response genes reveal signaling footprints in cancer gene expression. Nat Commun.

[42] Al-Zahrani KN, Abou-Hamad J, Cook DP, Pryce BR, Hodgins JJ, Labrèche C (2020). Loss of the Ste20-like kinase induces a basal/stem-like phenotype in HER2-positive breast cancers. Oncogene.

[43] Pryce BR, Al-Zahrani KN, Dufresne S, Belkina N, Labrèche C, Patino-Lopez G (2017). Deletion of the Ste20-like kinase SLK in skeletal muscle results in a progressive myopathy and muscle weakness. Skelet Muscle.

[44] Pryce BR, Labrèche C, Hamoudi D, Abou-Hamad J, Al-Zahrani KN, Hodgins JJ (2021). Muscle-specific deletion of SLK/Stk2 enhances p38 activity and myogenesis in mdx mice. Biochim Biophys Acta Mol Cell Res..

[45] González-González L, Alonso J (2018). Periostin: a matricellular protein with multiple functions in cancer development and progression. Front Oncol.

[46] Sarkar DK, Kim KH, Minami SJ (1992). Transforming growth factor-beta 1 messenger RNA and protein expression in the pituitary gland: its action on prolactin secretion and lactotropic growth. Mol Endocrinol.

[47] Ferrara N, Schweigerer L, Neufeld G, Mitchell R, Gospodarowicz D (1987). Pituitary follicular cells produce basic fibroblast growth factor. Proc Natl Acad Sci USA.

[48] Trowell O, Willmer EJ (1939). Studies on the growth of tissues in vitro: VI. the effects of some tissue extracts on the growth of periosteal fibroblasts. J Exp Biol.

[49] Armelin HA (1973). Pituitary extracts and steroid hormones in the control of 3T3 cell growth. Proc Natl Acad Sci USA.

[50] Gospodarowicz D (1975). Purification of a fibroblast growth factor from bovine pituitary. J Biol Chem.

[51] Lemmon SK, Bradshaw RA (1983). Purification and partial characterization of bovine pituitary fibroblast growth factor. J Cell Biochem.

[52] Babina IS, Turner NC (2017). Advances and challenges in targeting FGFR signalling in cancer. Nat Rev Cancer.

[53] Zhang Y, Alexander PB, Wang XF (2017). TGF-β family signaling in the control of cell proliferation and survival. Cold Spring Harb Perspect Biol.

[54] Ratajczak-Wielgomas K, Grzegrzolka J, Piotrowska A, Gomulkiewicz A, Witkiewicz W, Dziegiel P (2016). Periostin expression in cancer-associated fibroblasts of invasive ductal breast carcinoma. Oncol Rep.

[55] Xu J, Acharya S, Sahin O, Zhang Q, Saito Y, Yao J (2015). 14-3-3ζ turns TGF-β's function from tumor suppressor to metastasis promoter in breast cancer by contextual changes of Smad partners from p53 to Gli2. Cancer Cell.

[56] Ye X, Tam WL, Shibue T, Kaygusuz Y, Reinhardt F, Ng Eaton E (2015). Distinct EMT programs control normal mammary stem cells and tumour-initiating cells. Nature.

[57] Qi L, Zhou B, Chen J, Hu W, Bai R, Ye C (2019). Significant prognostic values of differentially expressed-aberrantly methylated hub genes in breast cancer. J Cancer.

[58] Levine DA, Bogomolniy F, Yee CJ, Lash A, Barakat RR, Borgen PI (2005). Frequent mutation of the PIK3CA gene in ovarian and breast cancers. Clin Cancer Res.

[59] Verret B, Cortes J, Bachelot T, Andre F, Arnedos M (2019). Efficacy of PI3K inhibitors in advanced breast cancer. Ann Oncol.

[60] Lindsley A, Snider P, Zhou H, Rogers R, Wang J, Olaopa M (2007). Identification and characterization of a novel Schwann and outflow tract endocardial cushion lineage-restricted periostin enhancer. Dev Biol.

[61] Kongkavitoon P, Butta P, Sanpavat A, Bhattarakosol P, Tangtanatakul P, Wongprom B (2018). Regulation of periostin expression by Notch signaling in hepatocytes and liver cancer cell lines. Biochem Biophys Res Commun.

[62] Hamidi A, Song J, Thakur N, Itoh S, Marcusson A, Bergh A (2017). TGF-β promotes PI3K-AKT signaling and prostate cancer cell migration through the TRAF6-mediated ubiquitylation of p85α. Sci Signal.

[63] Liu WT, Huang KY, Lu MC, Huang HL, Chen CY, Cheng YL (2017). TGF-β upregulates the translation of USP15 via the PI3K/AKT pathway to promote p53 stability. Oncogene.

[64] Yi JY, Shin I, Arteaga CL (2005). Type I transforming growth factor beta receptor binds to and activates phosphatidylinositol 3-kinase. J Biol Chem.

[65] Bakin AV, Tomlinson AK, Bhowmick NA, Moses HL, Arteaga CL (2000). Phosphatidylinositol 3-kinase function is required for transforming growth factor beta-mediated epithelial to mesenchymal transition and cell migration. J Biol Chem.

[66] Lamouille S, Derynck R (2007). Cell size and invasion in TGF-beta-induced epithelial to mesenchymal transition is regulated by activation of the mTOR pathway. J Cell Biol.

[67] Remy I, Montmarquette A, Michnick SW (2004). PKB/Akt modulates TGF-beta signalling through a direct interaction with Smad3. Nat Cell Biol.

[68] Conery AR, Cao Y, Thompson EA, Townsend CM, Ko TC, Luo K (2004). Akt interacts directly with Smad3 to regulate the sensitivity to TGF-beta induced apoptosis. Nat Cell Biol.

[69] Xie F, Jin K, Shao L, Fan Y, Tu Y, Li Y (2017). FAF1 phosphorylation by AKT accumulates TGF-β type II receptor and drives breast cancer metastasis. Nat Commun.

[70] Zhou M, Sutliff RL, Paul RJ, Lorenz JN, Hoying JB, Haudenschild CC (1998). Fibroblast growth factor 2 control of vascular tone. Nat Med.

[71] Ortega S, Ittmann M, Tsang SH, Ehrlich M, Basilico C (1998). Neuronal defects and delayed wound healing in mice lacking fibroblast growth factor 2. Proc Natl Acad Sci USA.

[72] Homer-Bouthiette C, Doetschman T, Xiao L, Hurley MM (2014). Knockout of nuclear high molecular weight FGF2 isoforms in mice modulates bone and phosphate homeostasis. J Biol Chem.

[73] Montero A, Okada Y, Tomita M, Ito M, Tsurukami H, Nakamura T (2000). Disruption of the fibroblast growth factor-2 gene results in decreased bone mass and bone formation. J Clin Invest.

[74] Markwald RR, Moreno-Rodriguez RA, Ghatak S, Misra S, Norris RA, Sugi Y (2019). Role of periostin in cardiac valve development. Adv Exp Med Biol.

[75] Nikoloudaki G, Creber K, Hamilton DW (2020). Wound healing and fibrosis: a contrasting role for periostin in skin and the oral mucosa. Am J Physiol Cell Physiol.

[76] Duchamp de Lageneste O, Colnot C (2019). Periostin in bone regeneration. Adv Exp Med Biol..

[77] Kattla JJ, Carew RM, Heljic M, Godson C, Brazil DP (2008). Protein kinase B/Akt activity is involved in renal TGF-beta1-driven epithelial–mesenchymal transition in vitro and in vivo. Am J Physiol Renal Physiol.

[78] Hsu AH, Lum MA, Shim KS, Frederick PJ, Morrison CD, Chen B (2018). Crosstalk between PKCα and PI3K/AKT signaling is tumor suppressive in the endometrium. Cell Rep.

[79] Isakov N (2018). Protein kinase C (PKC) isoforms in cancer, tumor promotion and tumor suppression. Semin Cancer Biol.

[80] Namciu S, Lieberman MA, Stavnezer E (1994). Induction of the c-ski proto-oncogene by phorbol ester correlates with induction of megakaryocyte differentiation. Oncogene.

