# Supplementary figures and images for: Periostin gene expression in neu-positive breast cancer cells is regulated by a FGFR signaling cross talk with TGFβ/PI3K/AKT pathways
Source: Breast Cancer Res. 2021 Nov 22;23:107. doi: 10.1186/s13058-021-01487-8 (PMC8607680; doi:10.1186/s13058-021-01487-8)

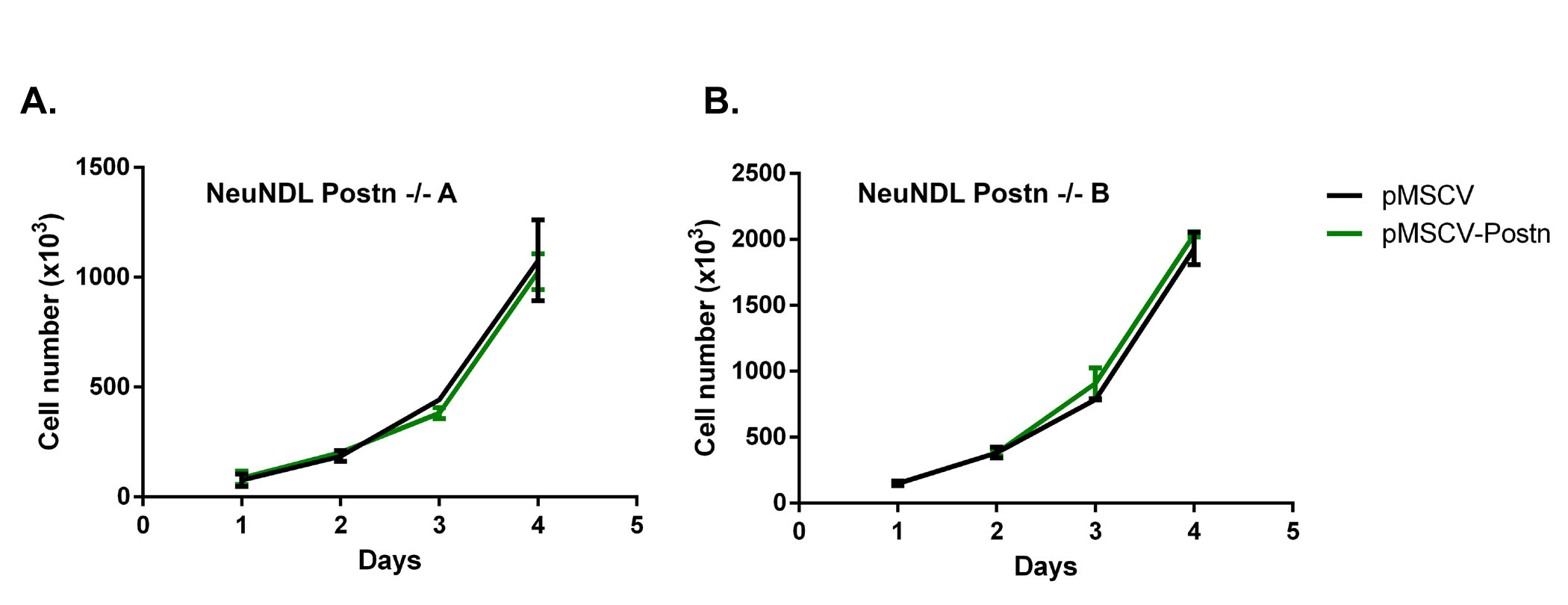

Supplement: Supplementary file 1 — Additional file 1: Figure S1. Growth rate is not affected in Postn-null breast cancer cells. NeuNDL Postn −/− cells were infected with retroviruses encoding Postn (pMSCV-Postn) or the empty vector (pMSCV). The cells were seeded and assessed for proliferation for up to 4-day post-seeding. This was performed on 2 independent isolates of Postn −/− cells (A and B). Data is represented as mean ± SEM. N = 3. [file 13058_2021_1487_MOESM1_ESM.jpg]

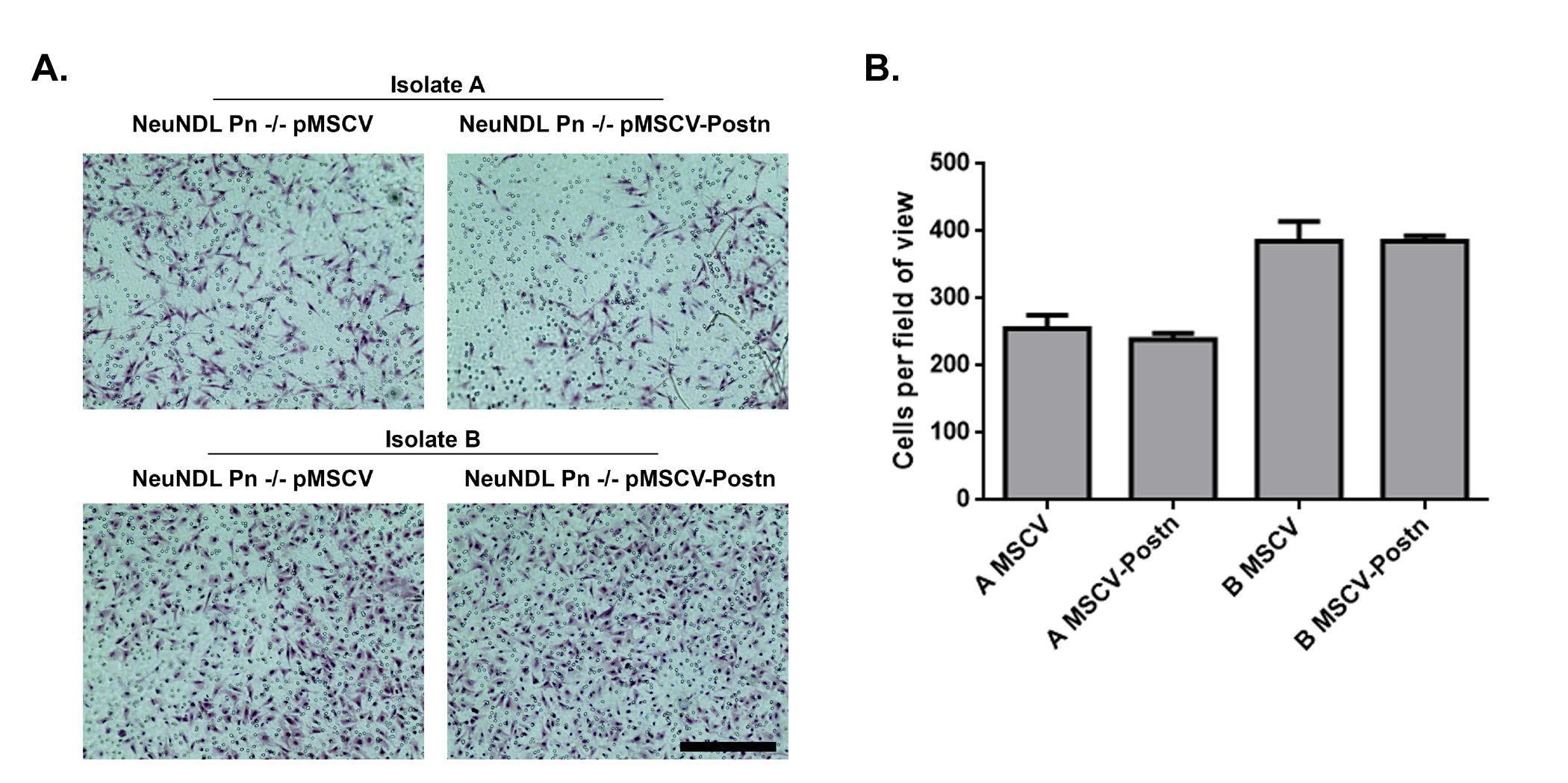

Supplement: Supplementary file 2 — Additional file 2: Figure S2. Migration rate is unaffected by forced expression of Postn. (A) Haptotaxis was assessed between NeuNDL Postn −/− cells stably re-expressing Postn (pMSCV-Postn) or the empty vector control (pMSCV). This was performed on 2 independent isolates of Postn −/− cells (A and B). Representative pictures of crystal violet stained membranes are shown. Scale bar = 200 µm. (B) Quantification by manual counting of 10 representative field of view per membrane is shown in the bar graph. Data is represented as mean ± SEM. N = 3. [file 13058_2021_1487_MOESM2_ESM.jpg]

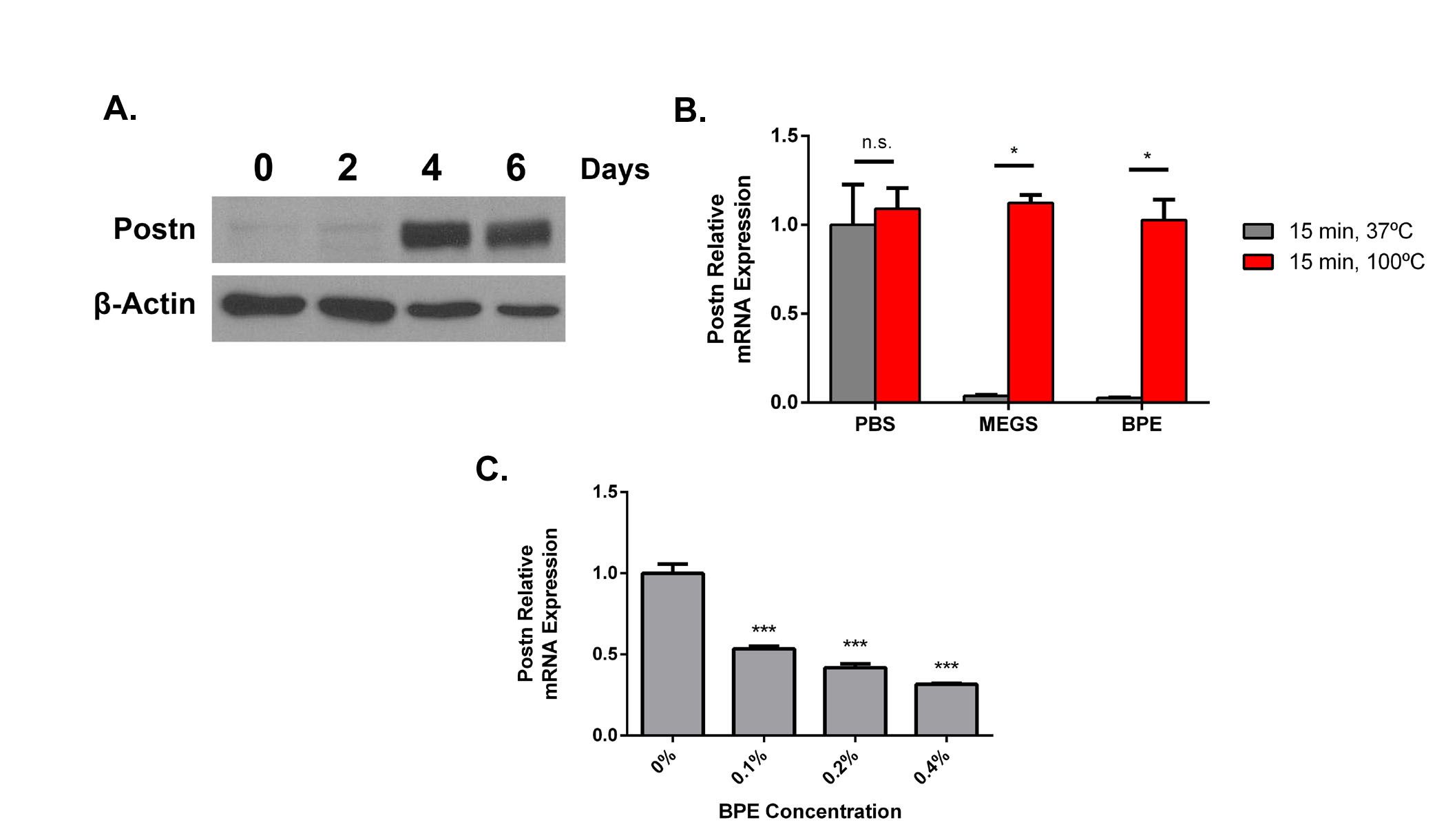

Supplement: Supplementary file 3 — Additional file 3: Figure S3. A short-lived heat labile BPE component represses Postn expression. (A) NeuNDL cells were left to grow in MEGS supplemented media and assessed for Postn protein expression after 2, 4 and 6 days without changing the medium. Upregulation of Postn suggests the depletion of a repressive activity. (B) NeuNDL cells were treated with PBS, MEGS and BPE were pre-incubated at 37°C or 100°C for 15 min to assess the heat sensitivity of the repressive components. Postn relative mRNA expression is shown in the bar graph for each condition. (C) NeuNDL cells were treated using 0%, 0.1%, 0.2% and 0.4% BPE to assess a dose-dependent relationship for the repression of Postn. Postn relative mRNA expression is shown in the bar graph for each condition. Data is represented as mean ± SEM. * = P ≤ 0.05 (C) [file 13058_2021_1487_MOESM3_ESM.jpg]

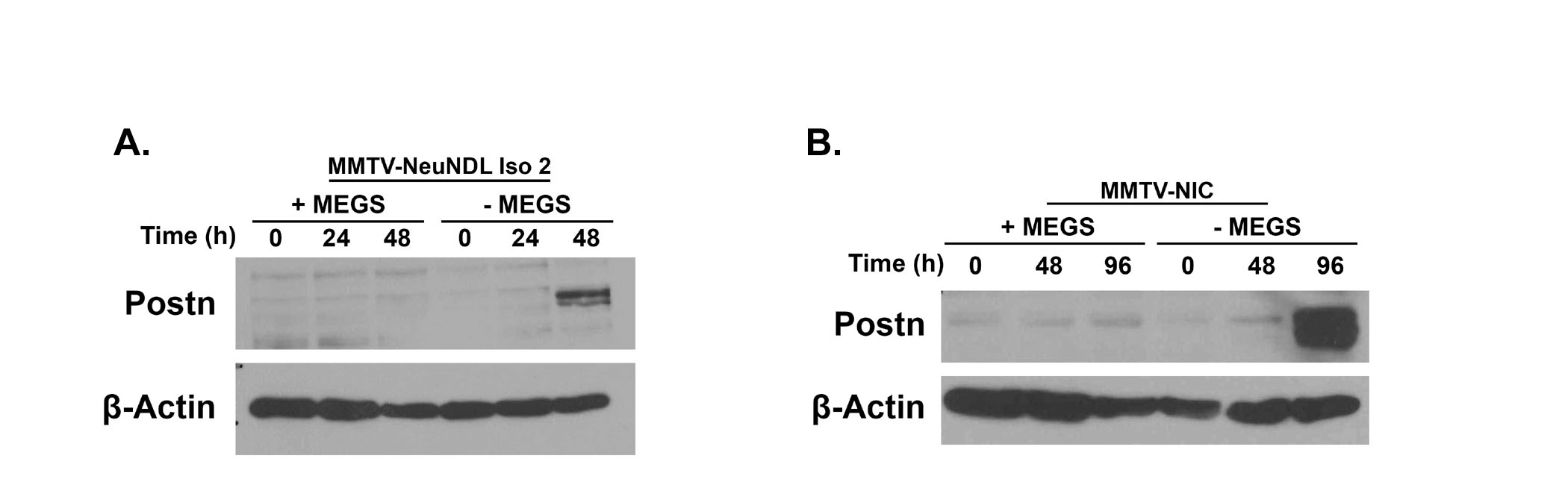

Supplement: Supplementary file 4 — Additional file 4: Figure S4. Removal of MEGS also induces Postn in other cell lines. An independent isolate (iso 2) of a MMTV-NeuNDL tumor (A) and an isolate from a MMTV-NIC tumor (B) were subjected to MEGS-deficient medium and Postn protein expression was assessed up to 96 h post-plating. [file 13058_2021_1487_MOESM4_ESM.jpg]

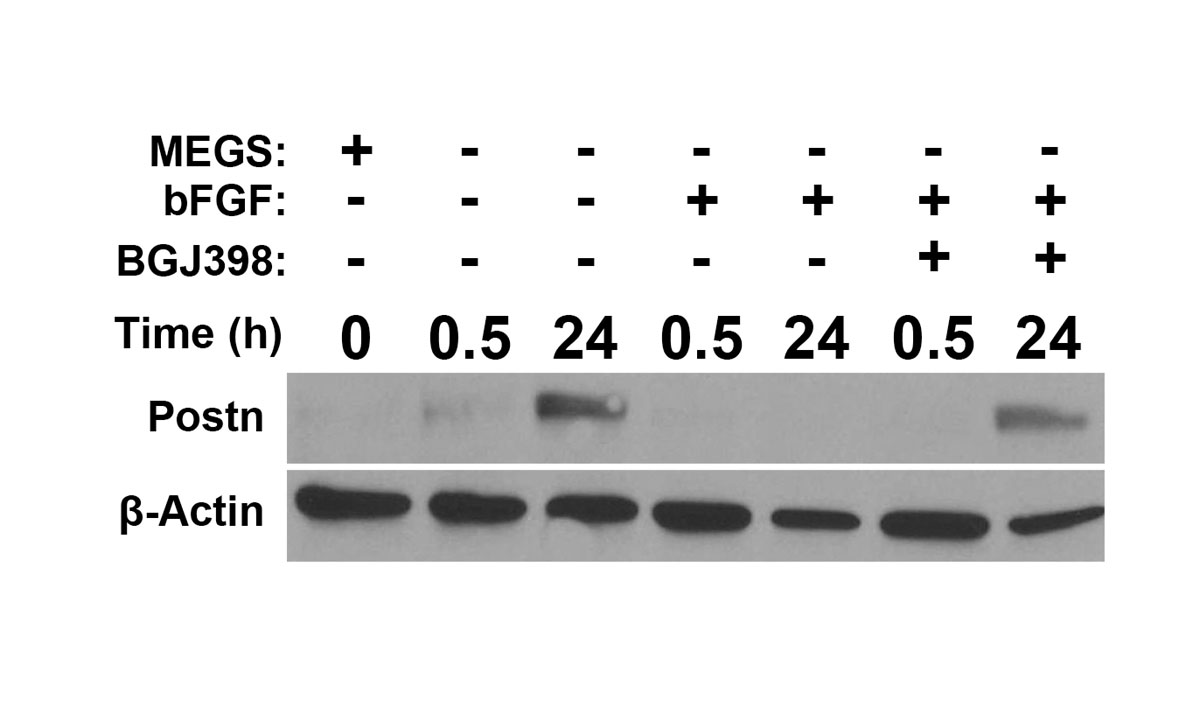

Supplement: Supplementary file 5 — Additional file 5: Figure S5. FGF2-mediated repression of Postn is rescued by an FGFR inhibitor. NeuNDL cells were plated in the absence of MEGS with 10ng/ml bFGF in combination with a pan-FGFR inhibitor BGJ398 at 100 nM. Postn protein expression was assessed by western blotting at 0.5- and 24-h post-treatment. [file 13058_2021_1487_MOESM5_ESM.jpg]

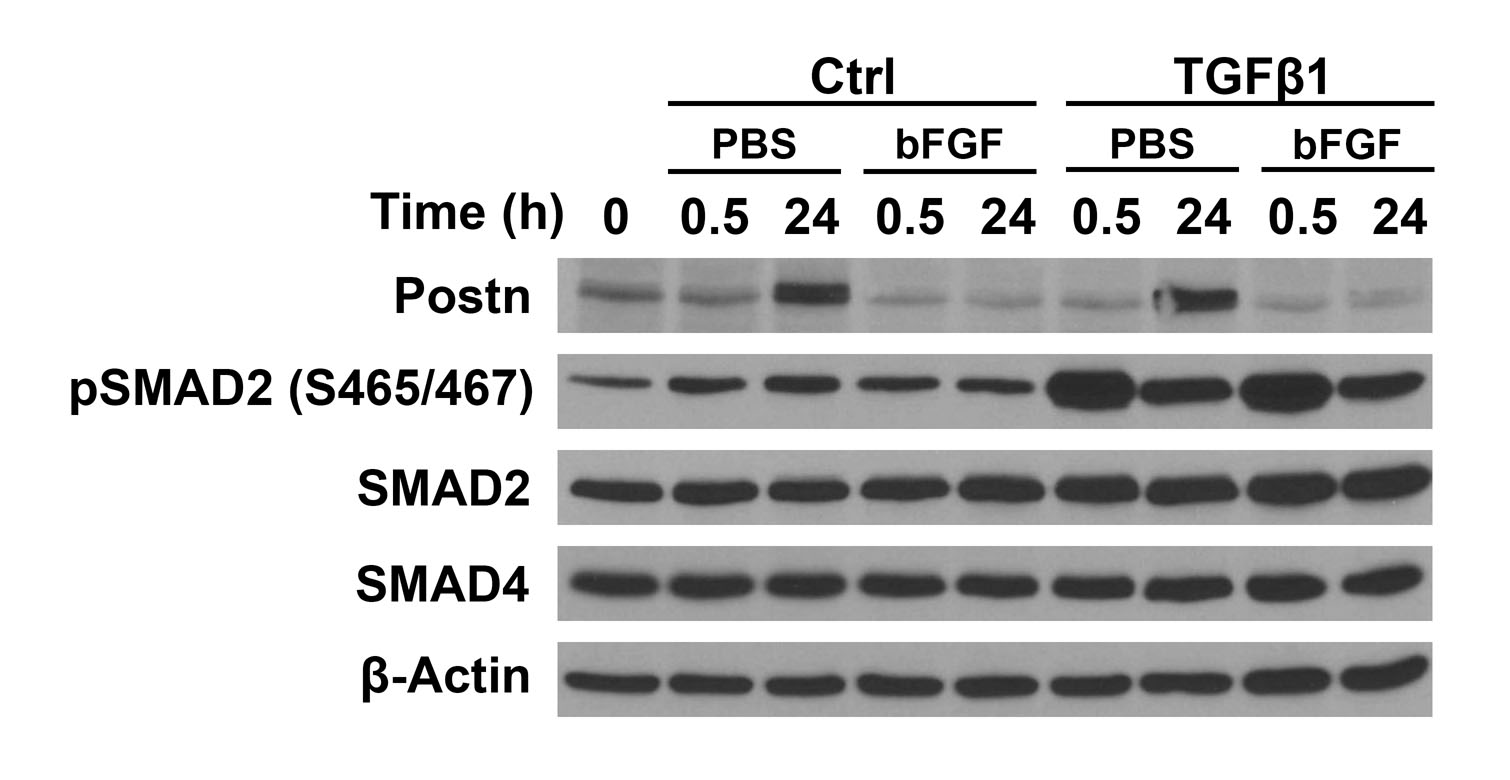

Supplement: Supplementary file 6 — Additional file 6: Figure S6. TGFβ-mediated induction of Postn is blocked by bFGF. NeuNDL cells were plated in the absence of MEGS and treated with 10 ng/ml of TGFβ-1 in combination with 10ng/ml of bFGF. Periostin protein expression was assessed 0.5- and 24-h post-treatment by western blotting analysis. Phospho-specific SMAD2 was used as a control for the activation of TGFβRs. [file 13058_2021_1487_MOESM6_ESM.jpg]

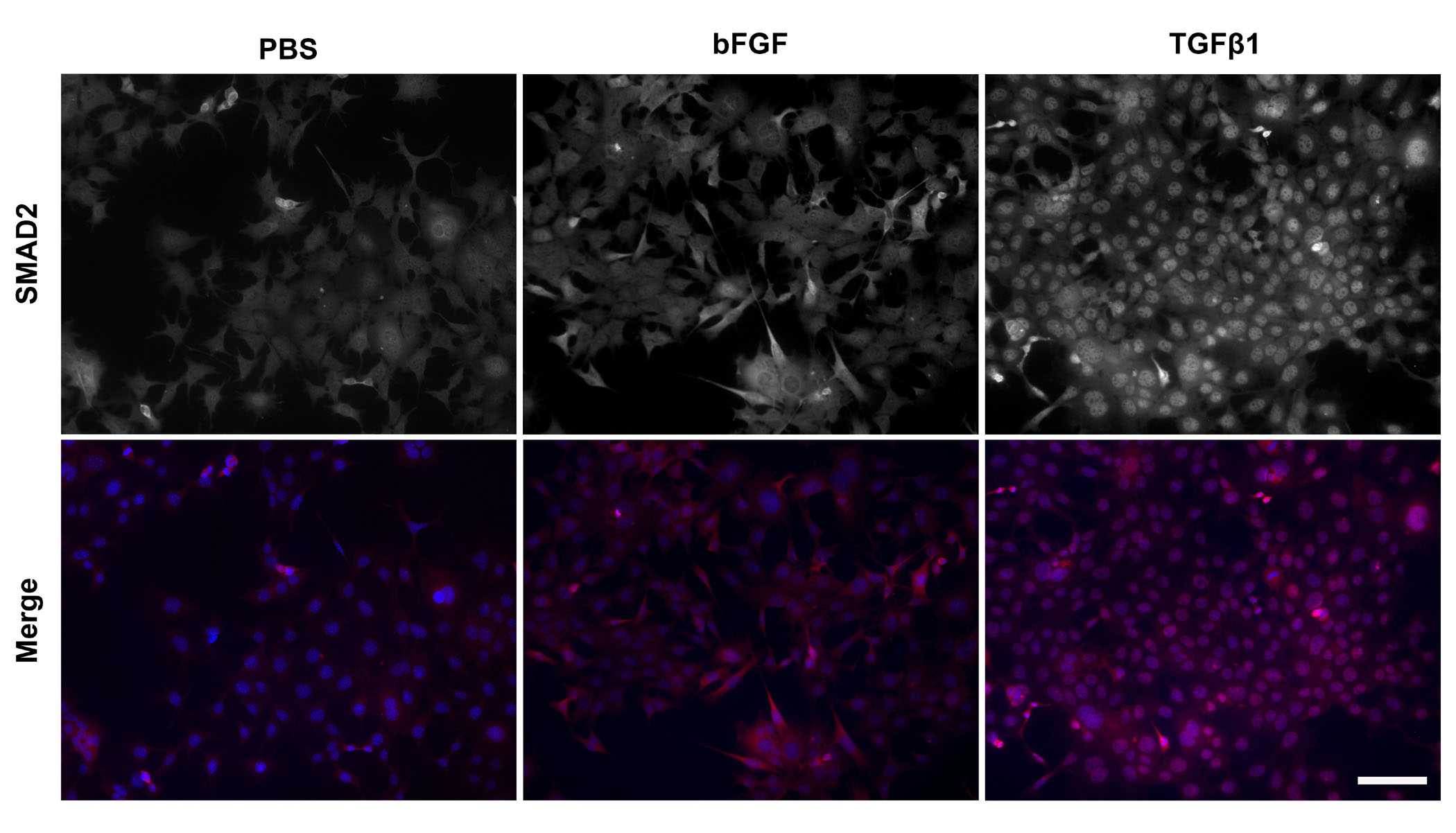

Supplement: Supplementary file 7 — Additional file 7: Figure S7. Postn induction is independent of SMAD2 activation. NeuNDL cells were subjected to MEGS-depleted conditions supplemented with either PBS, bFGF (10 ng/ml) or TGFβ-1 (10 ng/ml). Immunofluorescence using an anti-SMAD2 antibody is shown as a single channel and merged with DAPI. MEGS removal or FGF addition did not affect SMAD2 localization. Scale bar = 100 µm [file 13058_2021_1487_MOESM7_ESM.jpg]

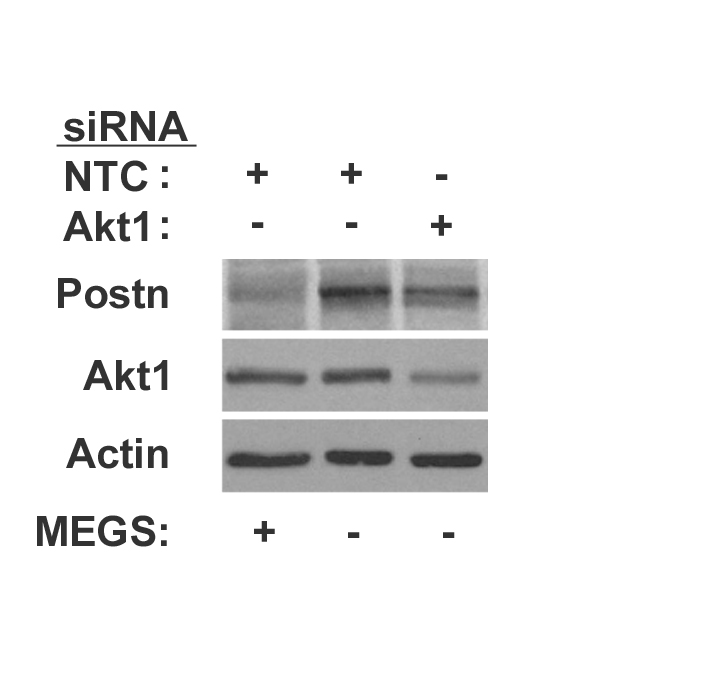

Supplement: Supplementary file 8 — Additional file 8: Figure S8. Akt1 knock down impairs Postn induction following MEGS depletion. Cultures were transfected with an Akt1 siRNA (or non-targeting control; NTC) in the presence of MEGS and then switched to MEGS depleted medium for 24 h. Postn and Akt1 expression were assessed by western blot. [file 13058_2021_1487_MOESM8_ESM.jpg]

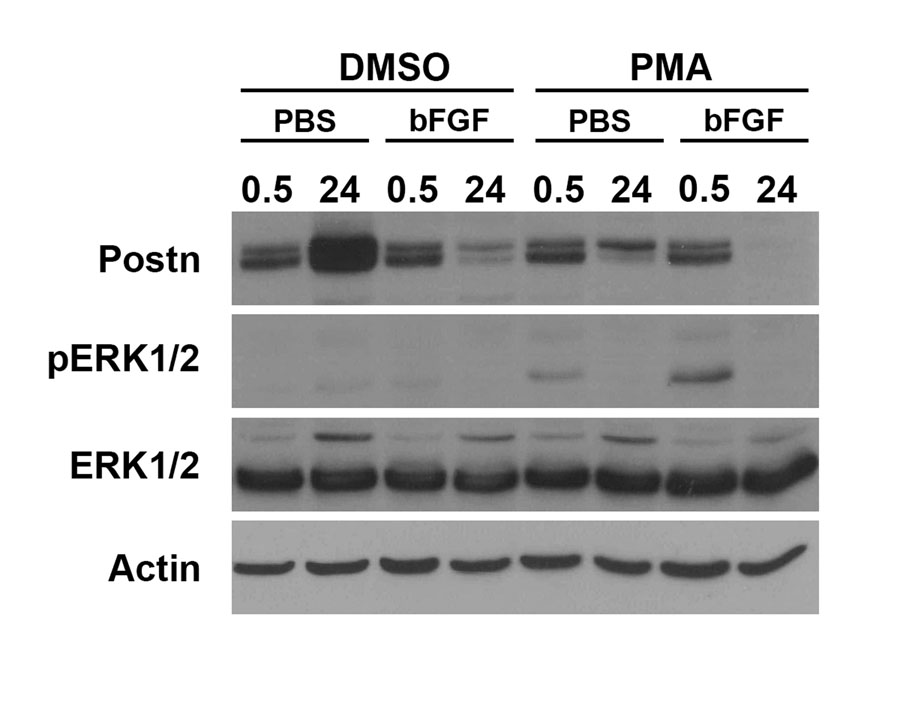

Supplement: Supplementary file 9 — Additional file 9: Figure S9. PKC activation can repress Postn expression and enhanced the effect of bFGF. NeuNDL cells were treated in absence of MEGS with PMA (1 µg/ml) in combination with bFGF (10 ng/ml). Postn expression was assessed by western blotting analysis and phospho-ERK1/2 was used as a control for PKC activation by PMA. [file 13058_2021_1487_MOESM9_ESM.jpg]
